# Postcranial anatomy of the Miocene hippopotamoids of Toros‐Menalla, Chad

**DOI:** 10.1111/joa.70135

**Published:** 2026-03-19

**Authors:** Lorenzo Scribano, Fabrice Lihoreau, Alexandra Houssaye, Clarisse Nekoulnang Djetounako, Jean‐Renaud Boisserie

**Affiliations:** ^1^ Institut des Sciences de l'Evolution de Montpellier, ISEM UMR CNRS 5554 Université Montpellier Montpellier France; ^2^ Département Adaptations du Vivant UMR 7179 CNRS/Muséum National d'Histoire Naturelle Paris France; ^3^ Centre National de Recherche pour le Développement N'Djamena Chad; ^4^ Centre Français des Études Éthiopiennes, UAR 3137 CNRS & Ambassade de France en Ethiopie Addis Ababa Ethiopia; ^5^ Laboratoire Paléontologie Évolution Paléoécosystèmes Paléoprimatologie, PALEVOPRIM UMR 7262 CNRS & Université de Poitiers Poitiers France

**Keywords:** Africa, Artiodactyla, Morphofunctional analysis, Paleoecology, Semi‐aquatic adaptations

## Abstract

Hippopotamoidea is a superfamily of cetartiodactyls that are nowadays limited to two extant species: *Hippopotamus amphibius*, the common hippopotamus, and *Choeropsis liberiensis*, the Liberian hippopotamus. These two mammals are endemic to Africa and inhabit ecosystems closely linked to water. They are the only extant members of a specialized ecological guild called the large semi‐aquatic herbivores. The majority of the diversity of this superfamily was composed by the paraphyletic anthracotheres, a geographically, temporally, and ecologically more diverse group from which hippopotamids likely originated. Historically, the phylogenetical relationships of these taxa were debated, especially since the establishment of the clade Cetancodonta which comprises cetaceans and hippopotamids. Research in this area has sought to address these issues using cranial, intracranial morphology, and dental anatomy. Their postcranial anatomy has until now been mostly unexploited data, sometimes due to poor preservation. At Toros‐Menalla (TM), a Late Miocene fossiliferous area in Chad, the last African anthracothere *Libycosaurus bahri* has been found coexisting with the large hippopotamid *Hexaprotodon garyam*. Their coexistence in humid environments suggests some form of niche‐partitioning. The locomotor apparatus is a significant means by which animals interact with their environment. It is a valuable resource for clarifying the phylogenetic issues previously cited, as well as for discussing functional and ecological considerations mentioned in previous literature. This study proposes an anatomical comparison between these two coexisting hippopotamoids and their closest extant ecomorph, the common hippopotamus. We have established a framework for the identification and differentiation of the postcranial skeleton of hippopotamoids by observing characters on a sample of approximately 650 specimens. We also highlight the importance of including the postcranial skeleton in future phylogenetical analyses. Additionally, we discuss the postcranial anatomy of those taxa in the context of the African Miocene environments, allowing new functional and ecological interpretations for the interaction between hippopotamoids and changes in their wet environments, which remain a major driver in the evolutionary history of these large artiodactyls.

## Graphical abstract

We establish a framework for the identification of postcranial remains in hippopotamoids by describing and comparing for the first time two late Miocene hippopotamoids from Toros‐Menalla, Chad (*Hexaprotodon garyam*, an early hippopotamid, and *Libycosaurus bahri*, the last African anthracothere) with the common hippopotamus. We highlight similarities between the two hippopotamids and a different morphology for the anthracothere, attributed to differences in behavior and in the exploitation of the late Miocene aquatic environments. We clarify the relevance of postcranial anatomy for future phylogenetic analyses and for the definition of Hippopotamoidea.

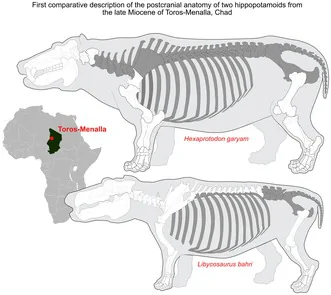

## 1 INTRODUCTION

Since the identification of cetaceans and hippopotamids as sister‐groups by molecular data (Montgelard et al., 1997; Tsagkogeorga et al., 2015; Waddell et al., 1999), the fossil record of these two vastly different and divergent groups has been the focus of vertebrate paleontologists. This clade, usually named Cetancodonta, is notably characterized by its dependence on water (Gatesy et al., 2013) that most probably corresponds to convergent evolution (Springer et al., 2021; Tsagkogeorga et al., 2015). Considering their fossil record, there is a consensus on the evolutionary history of cetaceans, including the progressive change in their aquatic behaviors (Cooper et al., 2011; Gatesy et al., 2013; Meloro, 2022; Pyenson, 2017; Thewissen et al., 2009; Werth et al., 2020). However, the early origin of hippopotamuses is less clear despite recent advances (Boisserie et al., 2005; Lihoreau et al., 2015; Orliac et al., 2010).

Hippopotamoidea is a superfamily of cetartiodactyls (Boisserie et al., 2011; Gentry & Hooker, 1988; Montgelard et al., 1997; Romer, 1966) which includes the hippopotamids and a vast paraphyletic group, the anthracotheres. The second group is more diverse due to a wider geographical, temporal and ecological extension (Lihoreau & Ducrocq, 2007). Hippopotamids are believed to have originated from these anthracotheres, most likely from an African lineage during the Oligocene (Boisserie et al., 2010; Lihoreau et al., 2015; Orliac et al., 2010).

In addition to the research conducted on the origin of the group, multiple projects were conducted to study the ecology of hippopotamoids (e.g., geochemistry, long bone microanatomy, inner‐ear study, dental wear: Boisserie, 2005; Boisserie & Merceron, 2011; Houssaye et al., 2021; Orliac et al., 2023). With the exception of mostly descriptive papers (Dineur, 1981; Ducrocq, 1999; Faure, 1983; Kowalewsky, 1873; Mazza, 1991; Miyamoto & Goodman, 1986; Pickford, 2015; Weston, 2000), most studies on hippopotamoids have left the postcranial skeleton untouched, mainly due to the difficulties of identification and access. Furthermore, the majority of these descriptive works did not incorporate discussions on phylogeny, ecology, or behavior (with the exception of Pickford, 2015), hindering a more comprehensive understanding of the biology and ecology of these animals.

In this study, we describe for the first time a uniquely well documented collection of two hippopotamoids from Chad. This material is attributed to one of the earliest hippopotamines (*Hexaprotodon garyam* Boisserie et al., 2005) and the latest African anthracothere (*Libycosaurus bahri* Lihoreau et al., 2014, belonging to the tribe Merycopotamini). By comparing them with the extant common hippopotamus, this study proposes an anatomical framework for the identification, differentiation and study of the postcranial skeletons of anthracotheres and hippopotamids. The primary objective of this comparison is to clarify the discriminating characters between two phylogenetically close taxa, also delving into their placement within Artiodactyla. With this exhaustive character investigation, we uncover new data on the postcranial anatomy of hippopotamoids, thereby helping to resolve taxonomical and anatomical issues that emerged in previous studies (Boisserie et al., 2005; Houssaye et al., 2021; Lihoreau et al., 2014), while also enhancing the accuracy of paleoecological and functional discussions and reconstructions. Finally, this study provides a new foundation for new phylogenetical insights brought by the incorporation of the postcranial skeleton in systematic analyses.

## 2 MATERIALS AND METHODS

### 2.1 Material

#### 2.1.1 Material provenience

The 575 fossil remains examined in this study were obtained from 149 fossiliferous localities within the Toros‐Menalla (TM) fossiliferous area in the Djurab Desert, situated in northern Chad (Le Fur et al., 2009). These specimens were collected by the Franco‐Chadian Paleoanthropological Mission (MPFT) between 1997 and 2018. The Anthracotheriid Unit, to which these Late Miocene localities belong, was biochronologically dated to between 7.4 Ma and 6.5 Ma (Vignaud et al., 2002), later restricted to between 7.2 Ma and 6.8 Ma based on authigenic ^10^Be/^9^Be dating (Lebatard et al., 2008). Each specimen is assigned an inventory number comprising the fossiliferous area prefix TM followed by the locality number, the year of excavation and a corresponding identification number (TMXXX‐YEAR‐NN).

**FIGURE 1:**
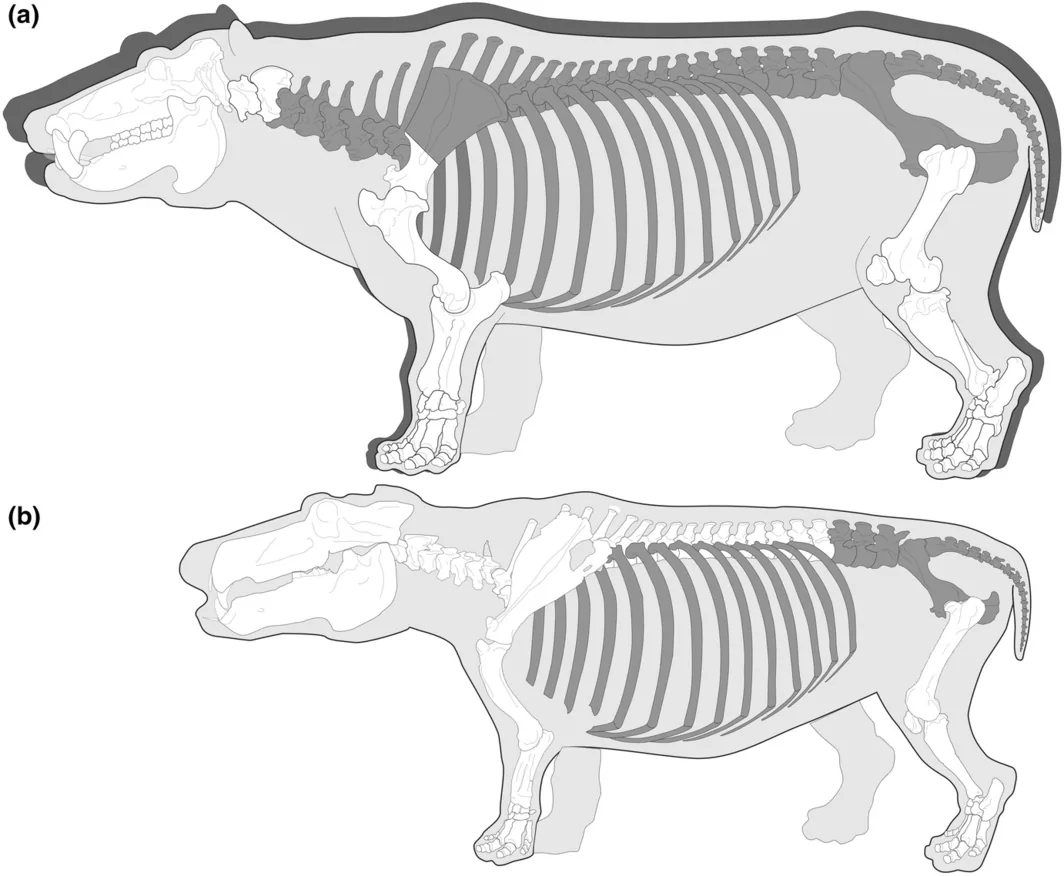
Vectorized silhouettes of the three hippopotamoids studied and availability of the postcranial material (adapted from archeozoo.org; Michel Coutureau, INRAP). (a) *Hippopotamus amphibius* in black and *Hexaprotodon garyam* in light grey; (b) *L. bahri*. In white known elements; in dark grey unknown elements.

All the postcranial specimens (Supplementary Material I) of Chadian hippopotamoids (Figure 1) are housed at the CNRD (Centre National de Recherche pour le Développement) in N'Djamena. The cranial and dental materials for both species have already been described (Boisserie et al., 2005; Lihoreau et al., 2006, 2014). The 216 specimens attributed to *Libycosaurus bahri* (*L. bahri*) represent one of the most substantial collections of postcranial remains for a single anthracothere species from a unique formation, with numerous complete bones and part of the skeleton found in anatomical position. Furthermore, this collection comes from a single stratigraphic unit, which is not the case for other large well‐known postcranial collections such as the early Oligocene deposits of the Egyptian Fayum (*Bothriogenys* sp.; Ducrocq, 1997) or the late early Oligocene of northern America (White River Formation; Prothero et al., 2022). A total of 359 specimens are referred to *He. garyam* (around three times as much as in Lothagam, Kenya, the now second largest Late Miocene collection in Africa).

#### 2.1.2 Comparative material

For the comparative material, we selected *Hippopotamus amphibius*, a close relative of *Hexaprotodon garyam*, belonging to the same subfamily Hippopotaminae. Notably, *Hi. amphibius* is the only extant hippopotamoid with abundant and available postcranial material. From an ecological standpoint, both *L. bahri* and *He. garyam* are regarded as close ecomorphs to *Hi. amphibius* (Boisserie et al., 2005; Lihoreau et al., 2014). This makes them relevant for comparative analyses of the taxa, enabling morphofunctional and phylogenetical interpretations. For this study, 141 specimens from at least 23 individuals were sampled, including both wild and captive specimens. These specimens all came from an array of European museums (see Supplementary I).

### 2.2 Methods

The anatomical framework for the nomenclature and orientation of bones follows previous work on common hippopotamus postcranial anatomy (Houtekamer & Sondaar, 1979; Walker, 1985; Weston, 1997). The nomenclature of the muscles, tendons, and ligaments is based on two studies on the limbs of extant hippopotamids (Fisher et al., 2007, 2010).

Measurements were obtained from either original specimens or pictures. A comparison of the methods revealed minimal error (measured on long bones with *n* = 36, mean measurement difference = −6.98 millimeters). The comparative material was measured during multiple separate missions by two different observers. Additionally, the specimens measured virtually are slightly overestimated, mainly due to the elevation of the scales compared to the measured bone. However, the average measurement error remains below the variation range of the bones studied. The different measurements were only taken when the specimens were complete or when the landmarks used for measuring a certain distance were present and identifiable in order to ensure their reliability (Supplementary Material II).

Multiple ratios were also calculated to facilitate morphological comparisons, such as those relating to differences in proportions (notably articular facet sizes).

Intralimb proportions of the forelimb and hindlimb were calculated using the length of the stylopod over the length of the zeugopod long bones. These ratios were calculated using means of lengths for each portion, as we lack complete individuals. However, the overall body size variation is weak among the three genera (see Supplementary Material II), allowing these ratios to be functionally interpreted.

The intra‐forelimb ratio (humeral length over radial length) was calculated using only the radial length and not the ulnar length as most of the weight is distributed on the radius in hippopotamoids, unlike in proboscideans (Bader et al., 2023; Mallet, 2020). The ratio between the proximal and the distal widths of the tibia was calculated in order to quantify the asymmetry of the tibia. In order to quantify compression or elongation in the unciform and the cuboid, we calculated the ratios between maximum height and width and between proximo‐distal width and transverse width, respectively.

Based on the calculations by Bader et al. (2023), the robustness of long bones was also calculated using the minimum diameter of the diaphysis over the maximum length of the bone (Ci/MaxL).

Body mass estimations for fossil specimens were based on astragali measurements following the regression devised by Martinez and Sudre (1995) (Y = 3.16 * X^1.248^, where Y is body mass in grams and X the product of length and width of the astragalus (L × l) measured in centimeters). Additional body mass estimations were calculated based on the long bones following the predictive equations of Scott (1983), although it is important to note that these equations were only based on bovids (Supplementary Material III).

The disto‐lateral curvature of the forelimb zeugopod was calculated graphically using the Affinity Designer software. The inverse of the radius of a circle was used to most closely fit the curve at the point where the curvature K is calculated (K = 1/R).

Most of the discriminant characters are illustrated on Figures 2, 3, 4, 5, 6, 7, 8, 9, 10, 11, 12, 13, 14. Reference to morphological traits on the figure and in the text are expressed as Nx with N being the abbreviation for the bone studied (i.e., Hm for humerus…) and x being the character number for this bone. The numbers reset for every bone (Supplementary Material I).

**FIGURE 2:**
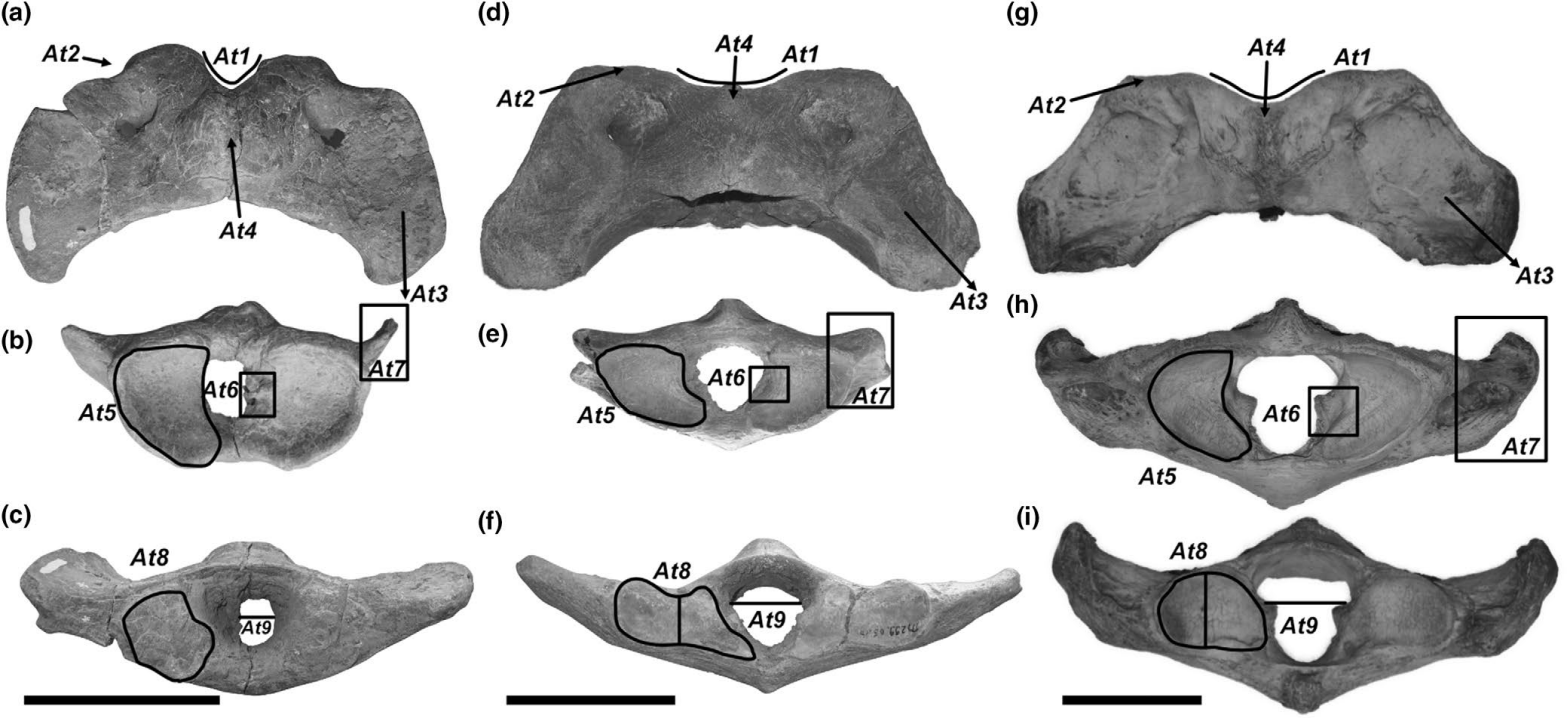
Atlas of hippopotamoids from TM and extant common hippopotamus. (a–c) *Libycosaurus bahri* (TM254‐04‐33) in dorsal (a), cranial (b) and caudal (c) views. (d–f) *Hexaprotodon garyam* (TM229‐05‐16) in dorsal (a), cranial (b) and caudal (c) views. (g–i) *Hippopotamus amphibius* (MNHN‐1897‐33) in dorsal (a), cranial (b) and caudal (c) views. Scale bars = 10 cm.

All measurements are described in the corresponding tab on the Excel sheet in supplementary data (Supplementary Material II).

## 3 RESULTS

### 3.1 Axial skeleton

#### 3.1.1 Atlas

In dorsal view, the cranial notch of *L. bahri* is narrow and deep (Figure 2a) in comparison to a shallower and wider condition seen in the hippopotamids (At1; Figure 2d,g). There is a marked notch on the cranial edge of the transverse process of the atlas (At2) in *L. bahri* (Figure 2a), unlike in *He. garyam* and *Hi. amphibius* where it is absent. *Hi. amphibius possesses* a thicker dorso‐ventral edge (Figure 2d,g). In *L. bahri*, the caudal shape of the transverse process (At3) is hook‐shaped and projected caudally, whereas in *He. garyam* and *Hi. amphibius* it is more rounded and projected latero‐caudally (Figure 2a,d,g). The spinous arch (At4) is cranially shifted in *Hi. amphibius* and *He. garyam* compared to a more central position in *L. bahri* (Figure 2a,d,g).

In cranial view, the cranial articular facets (At5) of *L. bahri* occupy a greater proportion of the total articular cranial surface area (Figure 2b). They proportionally take up less space in *He. garyam* and *Hi. amphibius* (Figure 2e,h).

The transition from the medial edge of the cranial facets to the neural canal (At6) is abrupt in *L. bahri* (Figure 2b). In contrast, this transition is smoother and more gradual in *He. garyam* and *Hi. amphibius* (Figure 2e,h). The cranial part of the transverse process (At7) is dorso‐ventrally short in *L. bahri*, is thick in *He. garyam* and even thicker in *Hi. amphibius* (Figure 2b,e,h).

In caudal view, the articular facets with the axis (At8) are rather round in shape and consist of a single facet in *L. bahri* (Figure 2c). They are subdivided into two joint facets oriented differently in *He. garyam* and *Hi. amphibius* and are elongated medio‐laterally in both, notably more so in *He. garyam* (Figure 2f,i). The vertebral canal of *Hi. amphibius* displays a moderate to marked T‐shaped opening (Figure 2i) bearing the odontoid apophysis of the axis. This opening is well less marked in *He. garyam* and absent in *L. bahri*, which displays a rounded canal (At9; Figure 2c,f).

#### 3.1.2 Axis

**FIGURE 3:**
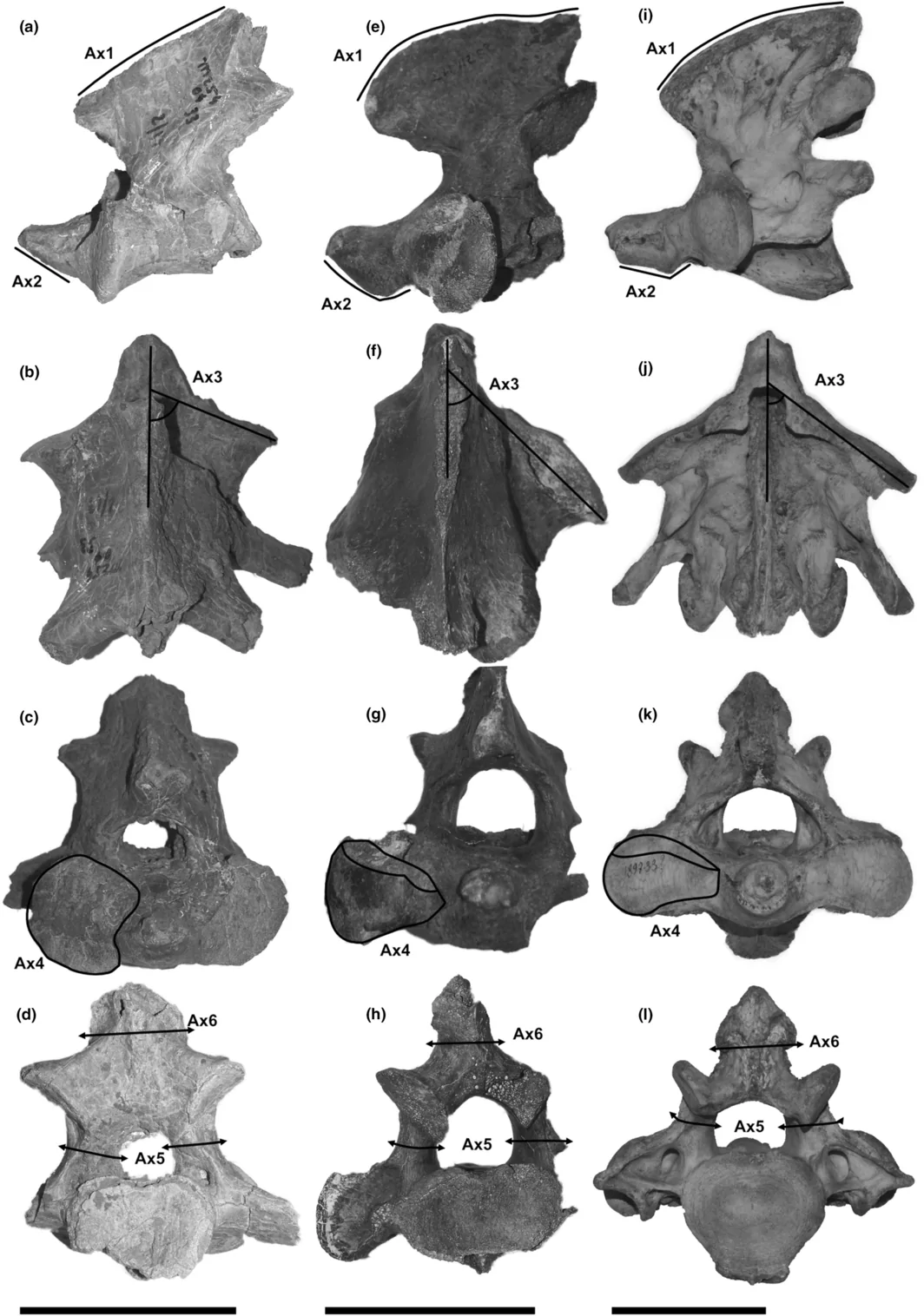
Axis of Hippopotamoids from TM and extant common hippopotamus. (a–d) *Libycosaurus bahri* (TM254‐04‐33) in lateral (a), dorsal (b), cranial (c), and caudal (d) views. (e–h) *Hexaprotodon garyam* (TM115‐06‐XX) in lateral (e), dorsal (f), cranial (g), and caudal (h) views. (i–l) *Hippopotamus amphibius* (MNHN‐1897‐33) in lateral (i), dorsal (j), cranial (k), and caudal (l) views. Scale bars = 10 cm.

In lateral view, the axis of *L. bahri* displays a rectilinear spinous process profile (Ax1; Figure 3a). This contrasts with the markedly convex profile in *Hi. amphibius* and in *He. garyam* (Figure 3e,i). The ventral side of the odontoid process (Ax2) is flat and ascending in *L. bahri* (Figure 3a). In both hippopotamids, the direction of the process is the same as in the merycopotamin, but it markedly protrudes ventrally in *He. garyam*, and slightly less in *Hi. amphibius* (Figure 3e,i).

The angle between the parasagittal plane of the axis and the prezygapophysis (Ax3) is greater than 60° in *L. bahri* (*n* = 1, Figure 3b) but is smaller than 60° in *Hi. amphibius* (*n* = 3) and *He. garyam* (*n* = 1) (Figure 3f,j). This correlates with the subdivision of the hippopotamids' caudal facets of the atlas (At8).

The articular surfaces of the prezygapophyses (Ax4) are more rounded in *L. bahri* (Figure 3c), displaying a single articular plane. In *He. garyam* and *Hi. amphibius* (Figure 3g,k), however, these surfaces are elongated and subdivided into dorsal and ventral articular facets. The neural arches (Ax5) are comparatively thicker and much more developed medio‐laterally in *L. bahri* (Figure 3d) than in both hippopotamids. The same trend is visible on the mediolateral thickness of the spinous process (Ax6; Figure 3h,f).

#### 3.1.3 Cervical vertebrae

It is important to note here the presence in TM of the only known complete cervical region from *Libycosaurus bahri* (C1‐C7, TM254‐04‐33).

**FIGURE 4:**
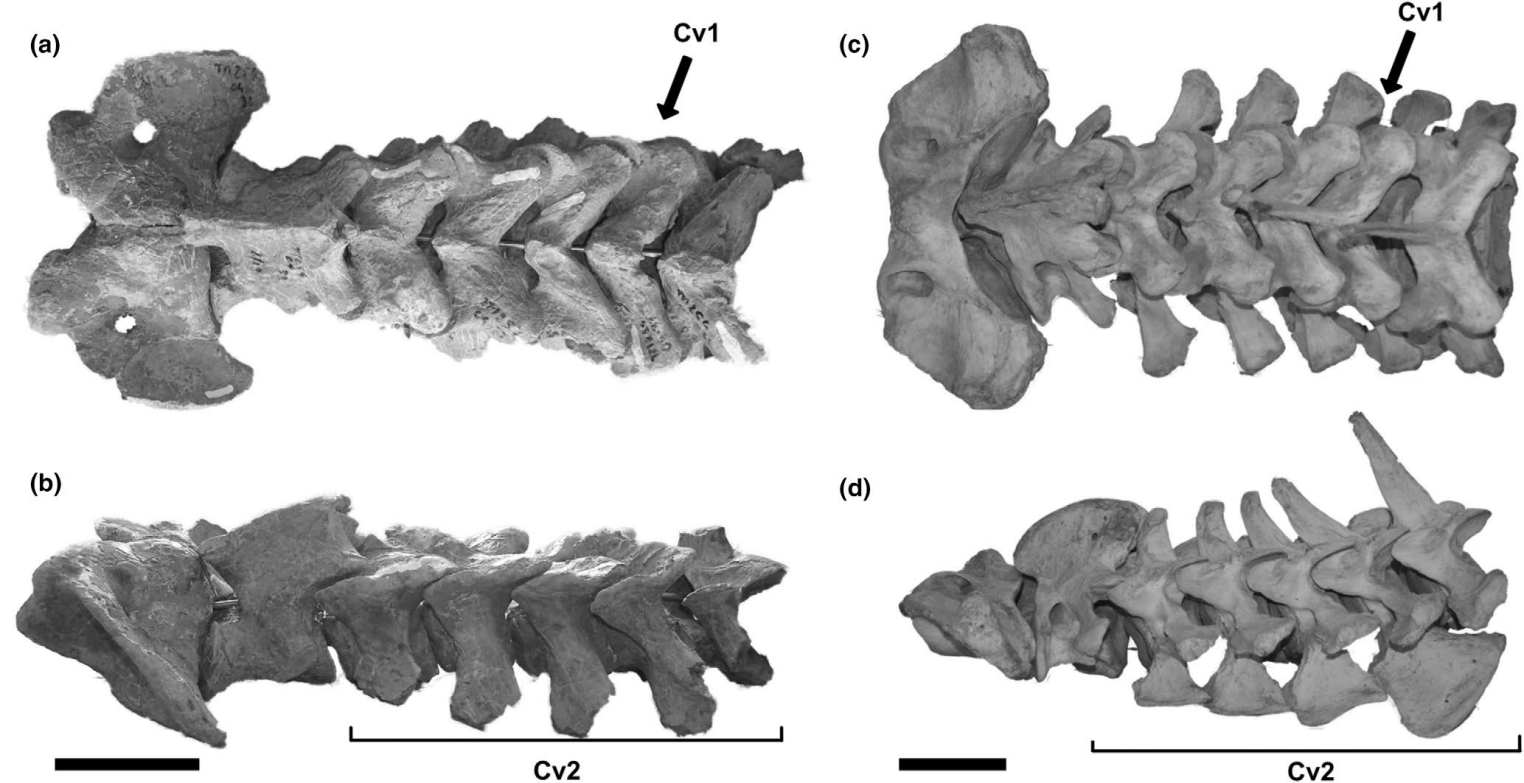
Cervical region of Hippopotamoids from TM and extant common hippopotamus. (a and b) *Libycosaurus bahri* (TM252‐04‐33) in dorsal (a) and lateral (b). (c and d) *Hippopotamus amphibius* (UM2) in dorsal (c) and lateral (d). Scale bars = 10 cm.

In dorsal view, the transverse processes (Cv1) are projected straight and perpendicular to the ground in *L. bahri* (Figure 4a). In contrast, they can be clearly seen in dorsal view and are thus projected more laterally in *Hi. amphibius* (Figure 4c).

In *Hi. amphibius*, additional transverse apophyses (Cv2) are strongly developed (Figure 4d). In both hippopotamoids, ventral parapophyses are visible. These are visibly less caudally enlarged in *L. bahri* than in *Hi. amphibius* (Figure 4b,d). They also increase in size in the latter, from the third cervical vertebra to the sixth, in contrast to what is observed in the merycopotamin (Figure 4b).

### 3.2 Girdles

Complete scapulas of hippopotamoids are rare in the fossil record, as most of the time only the proximal part can be found due to breakage (as seen in *He. garyam*, TM292‐09‐01, TM377‐04‐02). Nevertheless, a uniquely complete scapula of *L. bahri* has been retrieved and is studied here (TM266‐08‐48).

As for scapulas, complete pelvic bones are very rare in the fossil record of Hippopotamoidea. In TM, one fragmented pelvic bone, only comprised of the acetabulum, has been attributed to *L. bahri* (TM379‐04‐12b). The latter bone was found in connection to a proximal femur (TM379‐04‐12).

#### 3.2.1 Scapula

The overall shape of the scapula of *L. bahri* differs from that of *Hi. amphibius*, being more elongated cranio‐caudally and shorter dorso‐ventrally.

**FIGURE 5:**
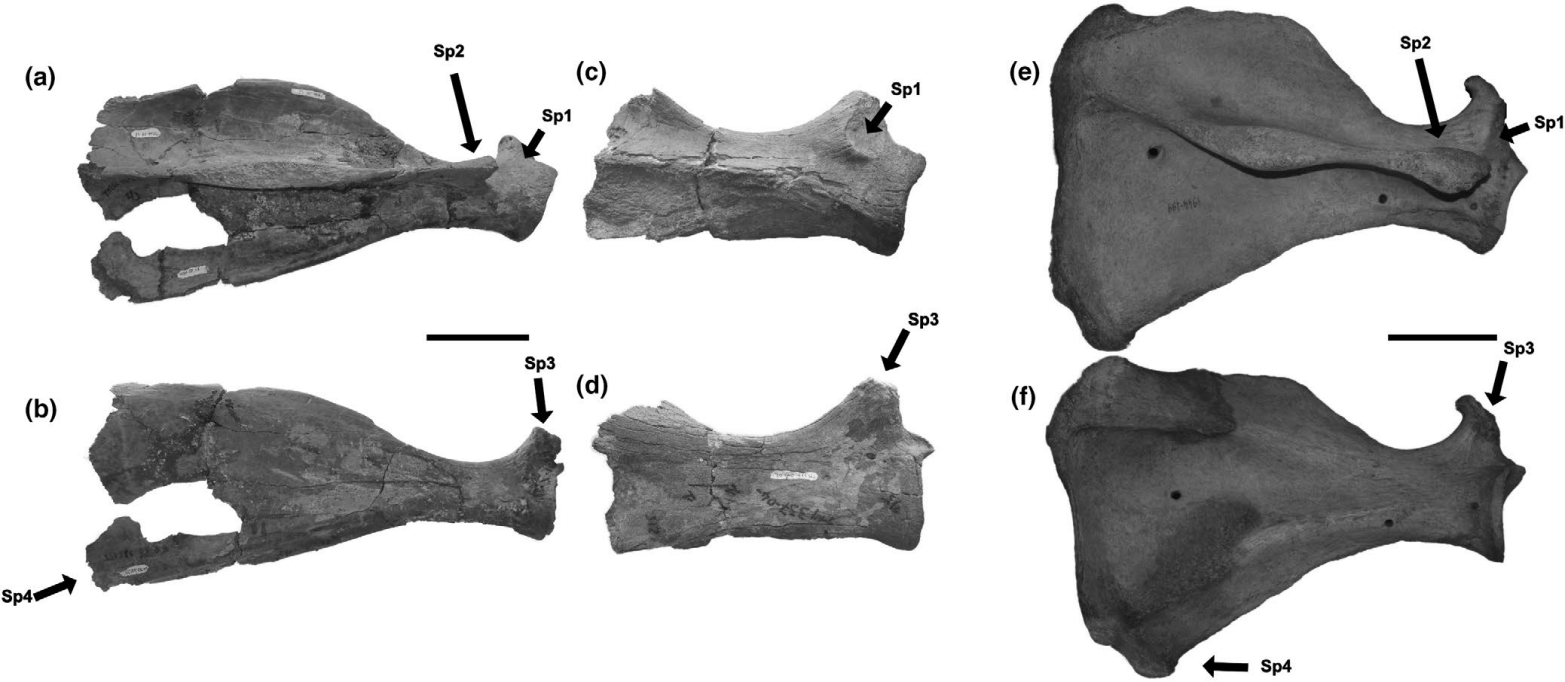
Scapula of Hippopotamoids from TM and extant common hippopotamus. (a and b) *Libycosaurus bahri* (TM226‐08‐48, right) in lateral (a) and medial (b, mirrored) views. (c and d) *Hexaprotodon garyam* (TM337‐04‐02, left) in lateral (c, mirrored) and medial (d) views. (e and f) *Hippopotamus amphibius* (MNHN‐1944‐199, right) in lateral (e) and medial (f, mirrored) views. Scale bars = 10 cm.

On the scapular neck, in lateral view, the biceps brachii muscle insertion area (Sp1) is weakly marked to absent in *L. bahri* (Figure 5a). Differently from the latter species, the hippopotamids present a clearly marked insertion of the same muscle (Figure 5d,g; Fisher et al., 2007).

The acromion process (Sp2) is more developed in *Hi. amphibius* than in *L. bahri* (Figure 5a,g). Unfortunately, it is not preserved in the specimen of *He. garyam*.

In *L. bahri*, the supraglenoid tubercle (Sp3) is positioned closer to the edge of the glenoid cavity (Figure 5b). This tubercle is, in medial view, slightly retreated dorsally in *He. garyam* and *Hi. amphibius* (Figure 5e,h).

The infraspinous fossa (Sp4) is more strongly developed in its dorsal part and more projected caudally in *Hi. Amphibius*, whereas it is less developed, with a weak caudal projection in *L. bahri* (Figure 5b,h). Sp4 is also not preserved in the specimen of *He. garyam*.

The glenoid cavity outline (Sp5) is oval and elongated dorso‐ventrally in *L. bahri* (Figure S1a) while it is more rounded in the hippopotamids (Figure S1b,c).

#### 3.2.2 Pelvic bone

The acetabulum of both *Hi. amphibius* and *L. bahri* is completely closed (Figure S2a,b ‐ Pv1) lacking a clearly recognizable acetabular notch.

### 3.3 Forelimb: Stylopod and zeugopod

There are multiple examinable humerus specimens for both extinct genera (respectively eight with five complete ones for *L. bahri* and 13 with four complete ones for *He. garyam*).

Regarding radioulnas, there are 23 specimens of *L. bahri*, including four complete bones. As for *He. garyam*, there are 25 specimens, among which six complete ones. The ratios for the intralimb proportions on the forelimb and for the robustness of both long bones are noted in Table 1.

**TABLE 1:** Ratios calculated for the three hippopotamoids, for better defining of characters.

Taxa	H/Ru	Robustness Humerus	Robustness Radioulna	F/T	Robustness Femur	Robustness Tibia	ProxW/DistW Tib	MaxH/MaxW Unc	CrW/TrW Cb
*Hippopotamus amphibius*	1.29	0.15	0.18	1.38	0.13	0.17	1.60	1.13	1.62
*Hexaprotodon garyam*	1.28	0.14	0.17	1.41	0.12	0.16	1.73	1.28	1.45
*Libycosaurus bahri*	1.51	0.14	0.18	1.32	0.12	0.15	1.30	1.13	1.40

Abbreviations: CrW/TrW Cb, Cranial cuboid width/Transverse cuboid width ratio; F/T, Femur/Tibia length ratio; H/Ru, Humerus/Radioulna length ratio; MaxH/MaxW Unc, Maximal unciform height/ Maximal unciform width ratio; ProxW/DistW Tib, proximal tibial width/distal tibial width ratio.

#### 3.3.1 Humerus

**FIGURE 6:**
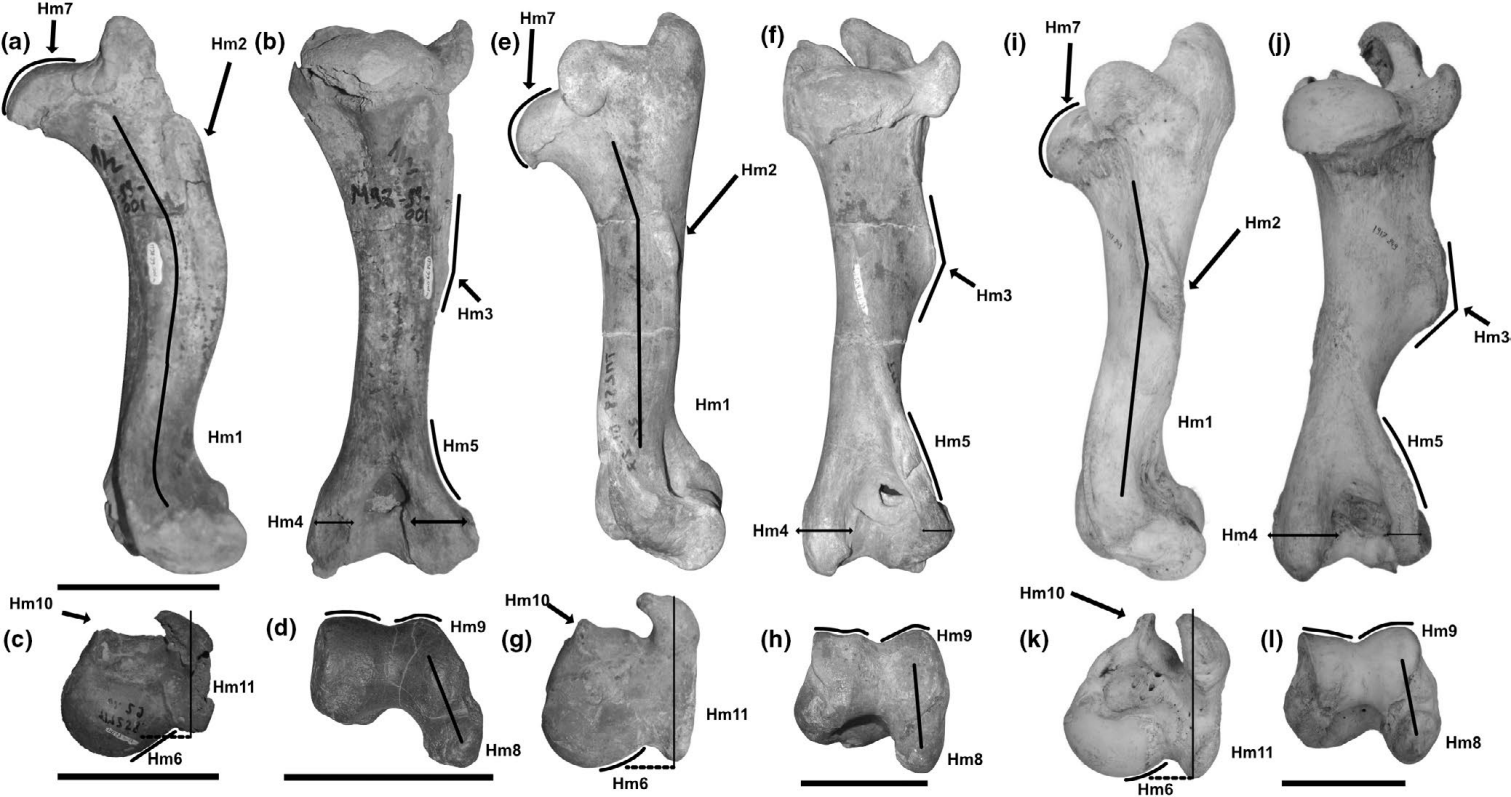
Humerus of Hippopotamoids from TM and extant common hippopotamus. (a–d) *Libycosaurus bahri* (TM98‐99–001, right for a/b; TM258‐01‐29, left for c, TM242‐04‐20, right for d) in lateral (a), caudal (b), proximal (c, mirrored), and distal (d) views. (e–h) *Hexaprotodon garyam* (TM258‐01‐27, left, mirrored) in lateral (e), caudal (f), proximal (g), and distal (h) views. (i–l) *Hippopotamus amphibius* (MNHN‐1917‐249, right) in lateral (i), caudal (j), proximal (k), and distal (l) views. Scale bars = 10 cm.

The humerus of *L. bahri* in lateral view shows a pronounced curvature of the rostral side (Hm1), forming a sigmoid curve and thus an S‐shaped bone (Figure 6a). In *He. garyam*, the caudal side of the humerus is straight, like in the extant common hippopotamus (Figure 6e,i).

The deltoid tuberosity is positioned (Hm2) at 2/5th of the total length of the bone in *Hi. amphibius* and *He. garyam* (Figure 6e,i), whereas it is more proximal, at around 1/5th from the proximal epiphysis in *L. bahri* (Figure 6a).

The deltoid tuberosity forms, in caudal view, a strong angle and is slightly projected laterally (Hm3) in *He. garyam* (Figure 6f). This is not the case in *L. bahri* where the tuberosity is projected cranially and forms a less marked angle (Figure 6b). *Hi. amphibius* (Figure 6f,j) displays a very strong lateral projection with an even more marked angle than in *He. garyam*.

The relative development of the epicondyles (Hm4) differs between the merycopotamin and the hippopotamids: The lateral epicondyle is more developed than the medial one in *L. bahri* (Figure 6b) whereas it is the reverse in *He. garyam* and *Hip. amphibius* (Figure 6b,f,j).

The supracondylar crest (Hm5) appears concave in *L. bahri* (Figure 6b) while it is convex in *Hi. amphibius* and *He. garyam* (Figure 6f,j).

In proximal view, the caudo‐lateral edge of the humeral head (Hm6) is straight in *L. bahri* whereas it is markedly curved in *He. garyam* and *Hip. amphibius* (Figure 6c,g,k). The articular surface of the humeral head (Hm7) is markedly projected caudally in lateral view in *L. bahri* (Figure 6a) whereas it is not in the hippopotamids (Figure 6e,i).

In distal view, the medial epicondyle (Hm8) is projected medio‐caudally in *L. bahri* (Figure 6d) whereas it is projected caudally in *He. garyam* and *Hip. amphibius* (Figure 6h,l). The ratio between the width of the capitulum and the trochlea (Hm9) is capitulum‐dominant in *L. bahri* (Figure 6d) and trochlear‐dominant to sub‐equal in hippopotamids (Figure 6h,l).

An inflated lesser tubercle (Hm10) is visible in *He. garyam* and *L. bahri* (Figure 6c,g), it is large even more developed in *Hi. amphibius* (Figure 6k).

The convexity of the major tubercle (Hm11) is extended caudally in the two hippopotamids (Figure 6g,k), reaching the caudal‐most point of the humeral head. In *L. bahri*, this convexity (Figure 6c) is not extended and stops at around 3/4th of the humeral head.

#### 3.3.2 Radioulna

**FIGURE 7:**
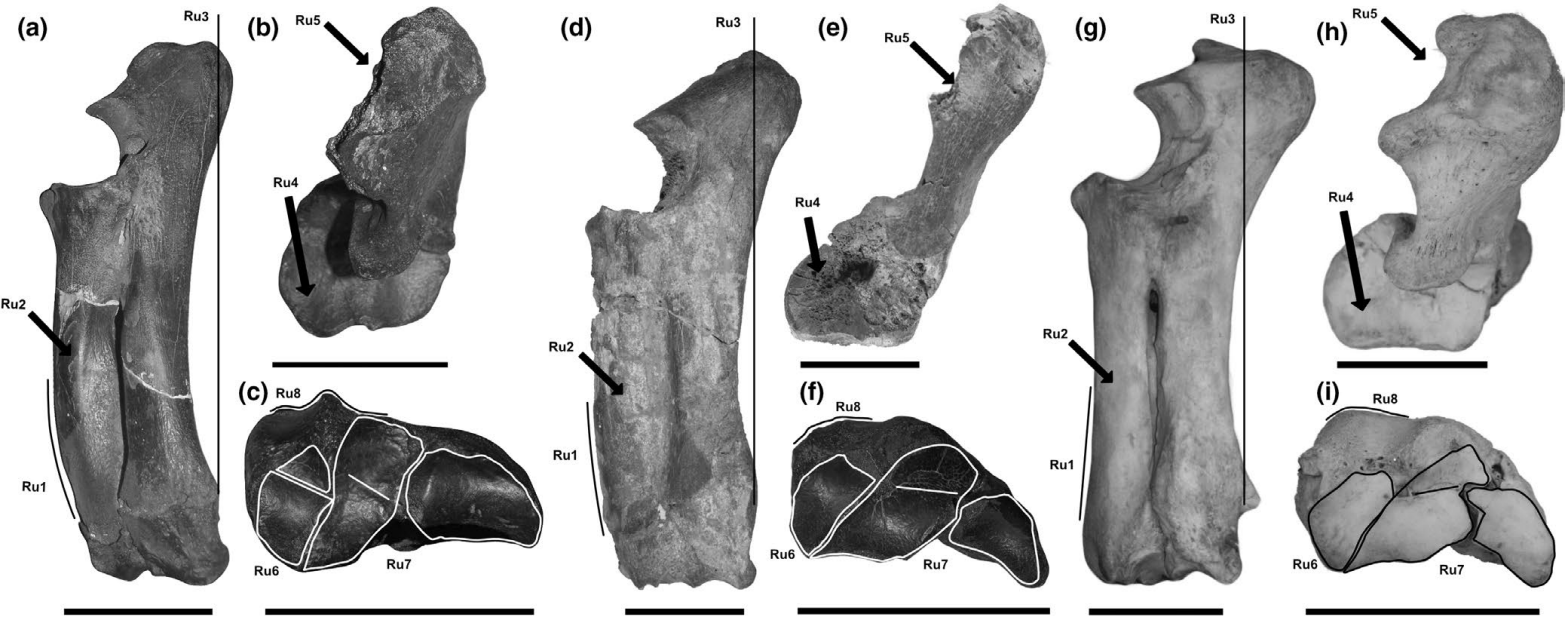
Radioulna of Hippopotamoids from TM and extant common hippopotamus. (a–c) *Libycosaurus bahri* (TM115‐00‐133, left) in lateral (a), proximal (b), and distal (c) views. (d–f) *Hexaprotodon garyam* (TM265‐08‐001, right for d/e; TM147‐01‐09, left for f) in lateral (d, mirrored), proximal (e, mirrored), and distal (f) views. (g–i) *Hippopotamus amphibius* (UM2, right for g; MNHN‐1917‐249, left for h/i) in lateral (g, mirrored), proximal (h), and distal (i) views. Scale bars = 10 cm.

In *L. bahri*, in lateral view, the distal part of the diaphysis of the radius (Ru1) is cranially convex whereas it is straight in *He. garyam* and *Hi. amphibius*. The general curvature of the radius is greater in *L. bahri* (Figure 7a; kLB = 0.021) than in the hippopotamids (Figure 7d,g; kHA = 0.003/kHG = 0.007).

A lateral ridge (Ru2) is marked on the diaphysis of the radius in *L. bahri* (Figure 7a) but is weak or absent in both hippopotamids (Figure 7d,g).

The caudal projection of the olecranon (Ru3) is not marked in *L. bahri* (Figure 7a). It is moderately strong in *He. garyam* (Figure 7d) and even more marked in *Hi. amphibius* (Figure 7g).

In proximal view, the anterior edge of the medial articular surface of the radius (Ru4) of *L. bahri* is protruding anteriorly (Figure 7b). In *Hi. amphibius* and *He. garyam* (Figure 7e, (h)), the profile of this anterior edge is flat. This is linked to the more significant trochlear development of the humerus in L. bahri (Hm4).

*L. bahri* and *He. garyam* (Figure 7b,e) display straight olecranon with no medial extension (Ru5) unlike *Hi. amphibius* (Figure 7h) that possesses a strong curved medial extension.

In distal view, the scaphoid‐radius contact (Ru6) differs between hippopotamids (Figure 7f, (i)), which possess a single articular facet, and *L. bahri* (Figure 7c) which displays two distinct articular facets separated by a groove, the cranial facet being smaller and oriented differently. In the radioulnar distal articular surface, the semilunar facet (Ru7) is the most prominent in *He. garyam* and *Hip. amphibius* (Figure 7f,i). In *L. bahri* (Figure 7c), semilunar and pyramidal articular facets are sub‐equal.

The cranio‐medial edge of the radius distal extremity (Ru8) displays a pointy‐shaped process in *L. bahri* (Figure 7c). This process is rounded and uniform in the hippopotamids (Figure 7f,i).

### 3.4 Forelimb: Carpals

The number of carpal bones available differs between the Chadian merycopotamin and the hippopotamids, with ten for *Hex*. garyam and six for *L. bahri*. All the carpal bones are nonetheless available for study for both extinct genera with the exception of the pyramidal, which is totally missing, and a single very damaged scaphoid of *L. bahri*. The ratio quantifying the compression of the unciform is noted in Table 1.

#### 3.4.1 Scaphoid

The scaphoid attributed to *L. bahri* is heavily damaged on both the proximal and distal articular surfaces and therefore does not allow us to be categorical on the observations made. Nevertheless, some characters are still clear on the dorsal and palmar edges.

**FIGURE 8:**
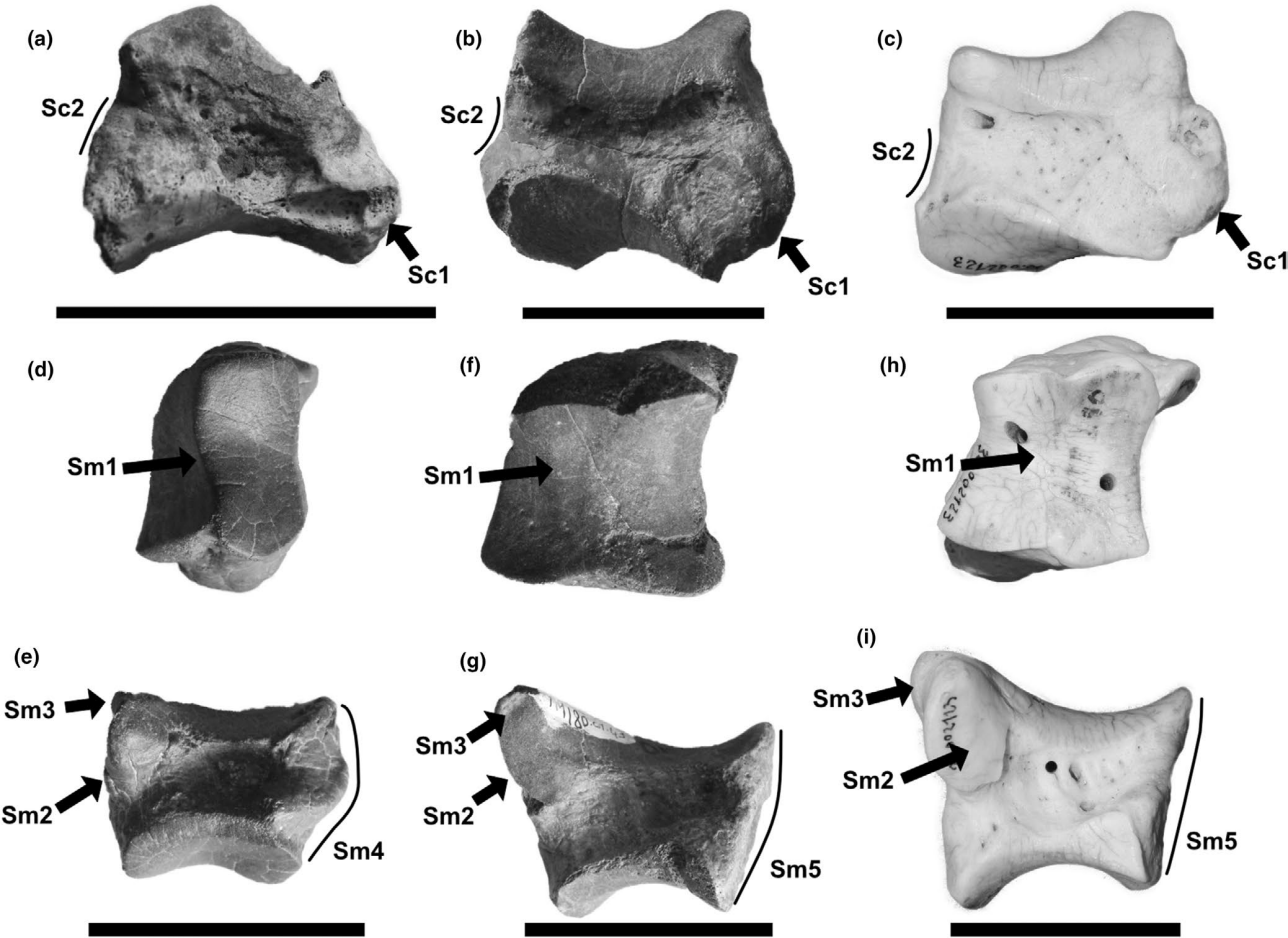
Proximal row of carpal bones of Hippopotamoids from TM and extant common hippopotamus. (a) Scaphoid; *Libycosaurus bahri* (TMX, right mirrored) in medial view (a). (b) Scaphoid; *Hexaprotodon garyam* (TM115‐09‐08, left) in medial view (b). (c) Scaphoid; *Hippopotamus amphibius* (OST‐361, left) in medial view (c). (d and e) Semilunar; *Libycosaurus bahri* (TM115‐09‐14, left) in distal (d) and lateral (e) views. (f and g) Semilunar; *Hexaprotodon garyam* (TM180‐01‐43, left) in distal (f) and lateral (g) views. (h and i) Semilunar; *Hippopotamus amphibius* (30.002123, left) in distal (h) and lateral (i) views. Scale bars = 5 cm.

In lateral view, the palmar process of the scaphoid (Sc1) is small to completely absent in *L. bahri* (Figure 8a), whereas it is present and represents around 1/4th of the bone in both hippopotamids (Figure 8b,c).

The dorsal edge of the scaphoid (Sc2) seems flat to lightly convex in *L. bahri* (Figure 8a). In contrast, it is markedly concave in the two hippopotamids (Figure 8b,c).

#### 3.4.2 Semilunar

In distal view, the angle formed by the two distal articular surfaces (Sm1) is way more acute in *L. bahri* (Figure 8d) than in the hippopotamids (Figure 8f,h).

The palmar edge of the semilunar (Sm2) is markedly convex in *L. bahri* (Figure 8e) in contrast to the straight to lightly convex edge in the hippopotamids (Figure 8g,i).

The lateral articular surface contacting the pyramidal (Sm3) looks similar in shape for *He. garyam* and *Hi. amphibius* (Figure 8g,i), being flat and developed proximo‐distally compared to *L. bahri* (Figure 8e), where it is rounder and more convex. This surface appears relatively larger in the hippopotamids (Figure 8h,i). In *L. bahri*, the cranio‐proximal semilunar process (Sm4) bearing the medial articular facet is much less cranially developed (Figure 8e) than in the hippopotamids (Figure 8g,i). The palmar edge of the pyramidal is markedly convex in *L. bahri* (Figure 8e). This convexity is light to absent in the hippopotamids (Figure 8g,i).

#### 3.4.3 Pyramidal

No pyramidal are attributable to *L. bahri*. Upon observation of the ulnar distal articulation, the pyramidal of the merycopotamin should be as large as the semilunar which is not the case in the hippopotamids of the sample.

The two hippopotamids are very similar in their pyramidal morphology. The only observable differences are in the contact facets with the semilunar (Py1/Py2). Medially, *Hi. amphibius* possesses relatively smaller contact facets compared to *He. garyam* (Figure S3a,b).

#### 3.4.4 Magnum

**FIGURE 9:**
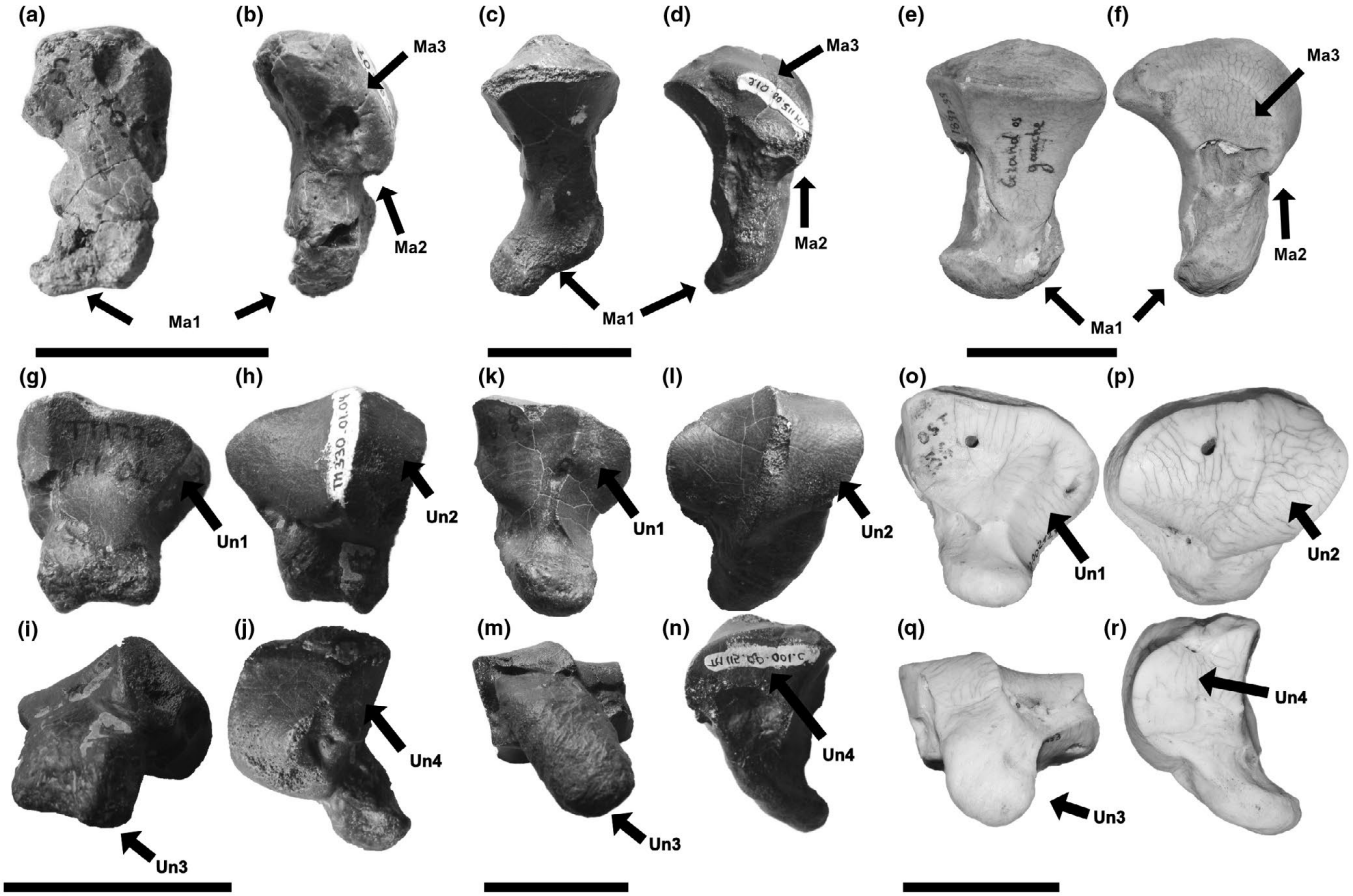
Distal row of carpal bones of Hippopotamoids from TM and extant common hippopotamus. (a and b) Magnum; *Libycosaurus bahri* (TM252‐05‐07, left) in palmar (a) and lateral (b) views. (c and d) Magnum; *Hexaprotodon garyam* (TM115‐08‐019, left) in palmar (c) and lateral (d) views. (e and f) Magnum; *Hippopotamus amphibius* (MNHN‐1897‐33, left) in palmar (e) and lateral (f) views. (g–j) Unciform; *Libycosaurus bahri* (TM330‐01‐04) in palmar (g), proximal (h), distal (i), and lateral (j) views. (k–n) Unciform; *Hexaprotodon garyam* (TM115‐00‐001c) in palmar (k, mirrored), proximal (l, mirrored), distal (m), and lateral (n, mirrored) views. (o–r) Unciform; *Hippopotamus amphibius* (OST‐361) in palmar (o), proximal (p), distal (q, mirrored), and lateral (r) views. Scale bars = 5 cm.

The palmar process of the magnum (Ma1) is globally thin and strongly projected caudally in *L. bahri* (Figure 9a). In *He. garyam*, it is relatively longer but retains a slender profile (Figure 9c). In *Hi. amphibius* (Figure 9e), this process is distinctly stronger and more caudo‐laterally projected.

The caudal notch under the articular surfaces with the semilunar and scaphoid (Ma2) is marked in *L. bahri* (Figure 9b). It is less marked to absent in *Hi. amphibius* and *He. garyam* (Figure 9d,f).

The lateral articular surface with the unciform (Ma3) is small and crescentic in *L. bahri* (Figure 9b). This facet is large and more globular in both hippopotamids (Figure 9d,f).

#### 3.4.5 Unciform

In disto‐palmar view, the facet for the metacarpal V (Un1) is relatively much smaller in *L. bahri* (Figure 9g) than in the two hippopotamids (Figure 9k,o).

The proximal surface comprises two articular facets: a medial facet for the semilunar and a lateral facet for the pyramidal. In the two hippopotamids (Figure 9l,p), the two facets (Un2) are similar in size whereas the semilunar facet appears significantly smaller in *L. bahri* (Figure 9h).

The palmar process (Un3) is thin, accounting for one third of the total width and tapers distally in *He. garyam* and *Hi. amphibius* (Figure 9m,q). In contrast, it is shorter and wider in *L. bahri* (Figure 9i).

In medial view, the semilunar facet (Un4) of *L. bahri* (Figure 9j) is slightly oriented medially. In the two hippopotamids (Figure 9n,r), it generally remains straight, more in line with the axis of the metacarpals. This results in less contact with the magnum in the merycopotamin than in *He. garyam* and *Hi. amphibius* (Figure 9j,n,r), in which the facet is well‐developed proximo‐distally. The ratio between the maximum height to the maximum width of the bone is approximately 1.13 for *L. bahri* and *Hi. amphibius* and approximately 1.28 for *He. garyam* (Figure 9h,l,p; Table 1).

### 3.5 Hindlimb: Stylopod and zeugopod

Femurs of *Hex. garyam* are more robust than those of *L. bahri*. A substantial number of specimens are available in TM, including eight complete specimens (nine with three complete specimens of *H. garyam* and 22 with five complete specimens of *L. bahri*).

Tibias are usually more fragmented. About 14 specimens are available for *L. bahri*, with only three complete and the rest being distal epiphyses. The sample for *Hex. garyam* comprises 74 specimens, 23 of which are complete and show different levels of wear.

The intralimb proportions ratios on the hindlimb and for the robustness of both long bones are shown in Table 1.

#### 3.5.1 Femur

**FIGURE 10:**
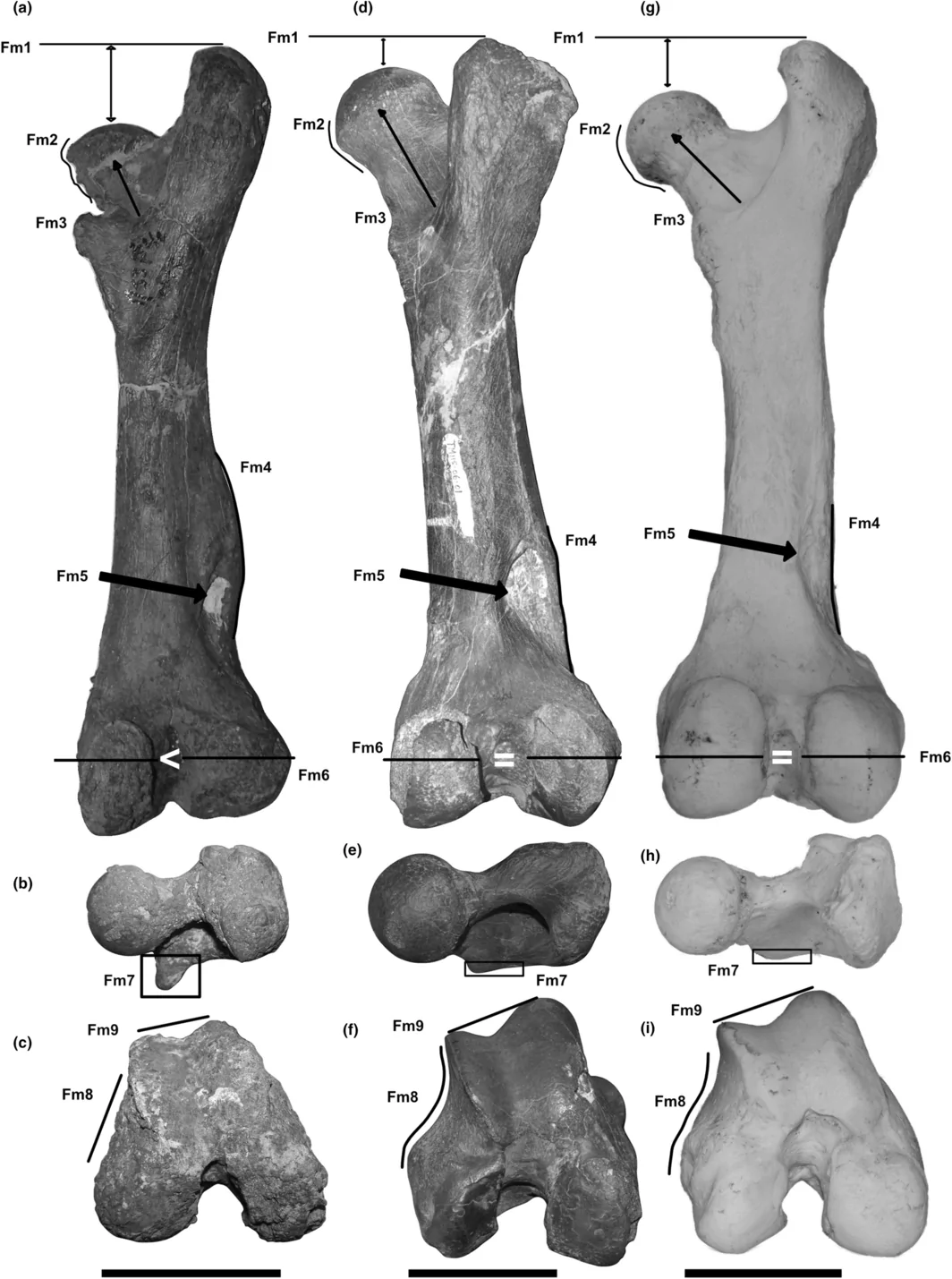
Femurs of Hippopotamoids from TM and extant common hippopotamus. (a–c) *Libycosaurus bahri* (TM53‐98‐12, right) in caudal (a), proximal (b), and distal (c) views. (d–f) *Hexaprotodon garyam* (TM115‐06‐01, right) in caudal (d), proximal (e), and distal (f) views. (g–i) *Hippopotamus amphibius* (MNHN‐1943‐27, right) in caudal (g), proximal (h), and distal (i) views. Scale bars = 10 cm.

The femur of *L. bahri* is slightly narrower than that of the hippopotamids (Table 1). The femoral head is more cranial in the former. The greater trochanter extends (Fm1) clearly proximally to the articular head in *L. bahri* (Figure 10a) but almost levels the head in *He. garyam* and *Hi. amphibius* (Figure 10d,h). On well‐preserved specimens, the disto‐lateral profile of the neck and head (Fm2) is oriented and projected proximally with a spherical shape in the hippopotamids whereas it is projected distally, thus hook‐shaped in *L. bahri* (broken in Figure 10a but well expressed on TM 112–04‐02). The femoral neck is shorter and thinner (Fm3) in *L. bahri* (Figure 10a) than in *He. garyam* and *Hi. amphibius* (Figure 10d,h).

The supracondylar fossa is marked in *L. bahri* (Figure 10a), extending above the midshaft (Fm5) and displaying a strong lateral projection (Fm4). In *Hi. amphibius* and *He. garyam* (Figure 10d,g), it is weakly projected laterally and is restricted to the distal part of the diaphysis. At the distal epiphysis, the lateral condyle is larger than the medial one (Fm6) in *L. bahri* (Figure 10a), whereas they are sub‐equal to slightly larger medially in *He. garyam* and *Hi. amphibius* (Figure 10d,h).

The three hippopotamoids lack the fovea capitis. The lesser trochanter (Fm7) is strongly projected medially in *L. bahri* (Figure 10b) but is limited to a weak tuberosity in *He. garyam and Hi. amphibius* (Figure 10e,h).

The lateral epicondyle, in distal view (Fm8), is markedly concave cranially and extended, forming a S‐shape outline in *He. garyam* (Figure 10f). It is quite similar in *Hi. amphibius* (Figure 10i), although less extended and flatter. In *L. bahri* (Figure 10c), there is no concavity, and the extension is very weak making the outline of the epicondyle straight. In *L. bahri* (Figure 10c), the lips of the trochlea (Fm9) are almost symmetrical. In both hippopotamids, there is a clearly stronger development and cranial projection of the medial lip of the trochlea (Figure 10f,i).

#### 3.5.2 Patella

On the patella, a large medial tuberosity (Pa1), extending the palmar articular surface, is visible only in hippopotamids (Figure S4a–c). The proximo‐dorsal edge of the patella (Pa2) is rounded in *L. bahri* (Figure S4a) and displays a more rectangular conformation in *He. garyam* and *Hi. amphibius* (Figure S4b,c).

#### 3.5.3 Tibia

**FIGURE 11:**
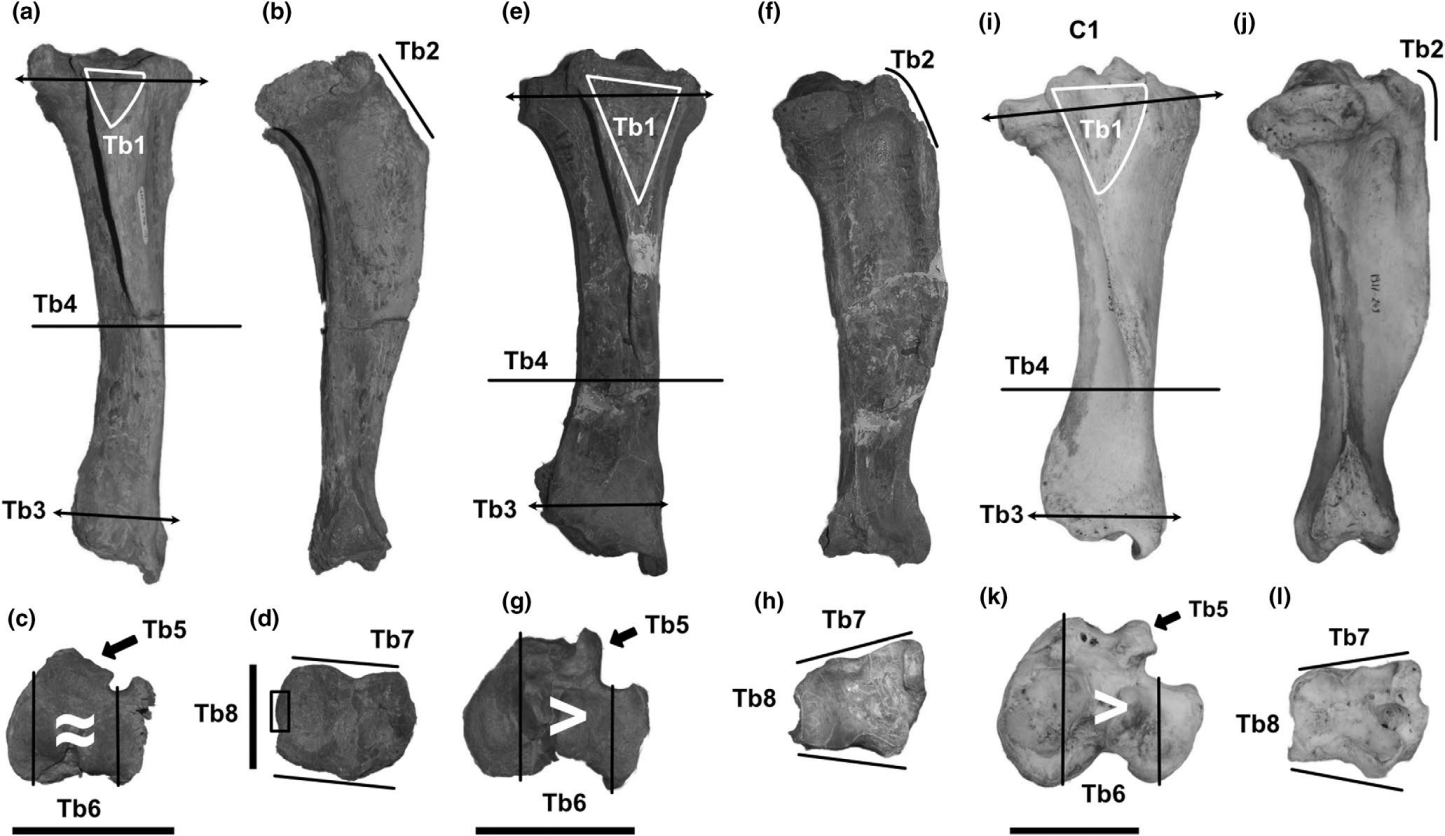
Tibias of Hippopotamoids from TM and extant common hippopotamus. (a–d) Tibia; *Libycosaurus bahri* (TM299‐09‐02, left for a–c; TM258‐04‐008, left for d) in cranial (a, mirrored), medial (b, mirrored), proximal (c, mirrored), and distal (d, mirrored) views. (e–h) Tibia; *Hexaprotodon garyam* (TM258‐04‐30, right for e/f/h; TM09‐01‐70, left for g) in cranial (e), medial (f), proximal (g, mirrored), and distal (h) views. (i–l) Tibia; *Hippopotamus amphibius* (MNHN‐1917‐249, right) in cranial (i), medial (j), proximal (k), and distal (l) views. Scale bars = 10 cm.

The patellar fossa is triangular in all three taxa, but varies in size, being relatively smaller in *L. bahri* (Figure 11a) than in *Hip*., *amphibius* and *He. garyam* (Tb1; Figure 11e,i). In medial and lateral views, the proximal part of the tibial crest (Tb2) is straighter and more proximally angled in *L. bahri (*Figure 11b) than in the hippopotamids (Figure 11f,j).

The tibial crest (Tb4) extends from the proximal epiphysis to midshaft in *L. bahri* (Figure 11a) while it constitutes approximately 75% of the diaphysis in the hippopotamids (Figure 11e,i).

The tibial tuberosity (Tb5) is less developed in *L. bahri* (Figure 11c) than it is in the hippopotamids (Figure 11g,k). The medial and lateral articular surfaces of the tibial plateau (Tb6) are of sub‐equal size in *L. bahri* (Figure 11c). There is a marked asymmetry, with the medial surface wider than the lateral one in *He. garyam* and *Hi. amphibius* (Figure 11g,k). This asymmetry is inverted compared to the one on the femur condyles (Fm6; Figure 11a,d,g).

In distal view (Tb7), the hippopotamids present a trapezoidal outline with an enlargement of the medial side (Figure 11h,l), whereas *L. bahri* presents a more rectangular outline (Figure 11d). A small articular surface for the fibula (Tb8) is also present on the lateral distal end of the tibia, but only in *L. bahri* (Figure 11d,h,l). The ratio between the transversal width of the proximal and distal ends of the tibia (Tb3) is greater in *Hi. amphibius* than in the fossil hippopotamoids (Figure 11a,e,i; Table 1).

### 3.6 Hindlimb: Tarsals

Our sample includes 236 tarsal bones, with the proximal row of the tarsals (astragali and calcanei) being much more represented (*n* = 220) than the distal row (cuboids and naviculars, *n* = 16).

*Hexaprotodon garyam* is represented by 165 tarsals with nine from the distal row. *Libycosaurus bahri* is represented by 71 tarsals with seven from the distal row.

The ratios calculating the compression of the cuboid are noted in Table 1.

#### 3.6.1 Astragalus,

**FIGURE 12:**
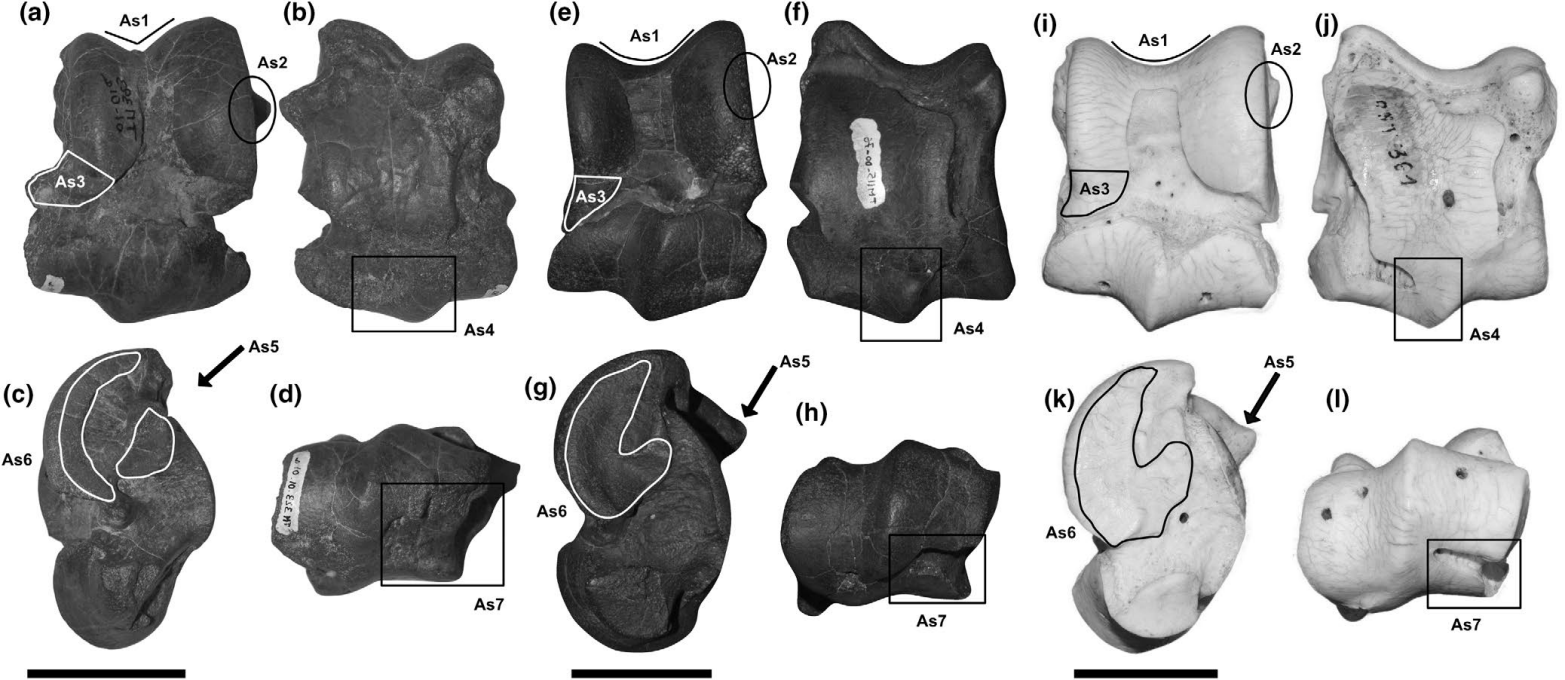
Astragali of Hippopotamoids from TM and extant common hippopotamus. (a–c) *Libycosaurus bahri* (TM323‐01‐01a, right, mirrored) in cranial (a), palmar (b), lateral (c), and distal (d) views. (d–f) *Hexaprotodon garyam* (TM115‐00‐76, right, mirrored) in cranial (e), palmar (f), lateral (g), and distal (h) views. (g–i) *Hippopotamus amphibius* (OST‐361, left) in cranial (i), palmar (j), lateral (k), and distal (l) views. Scale bars = 5 cm.

The proximal trochlea (As1) is V‐shaped in *L. bahri* (Figure 12a), whereas it is broad and U‐shaped in the hippopotamids (Figure 12e,i). The lateral malleolar process of the calcaneus, on the lateral side of the proximal trochlea (As2), is protruding in *L. bahri* (Figure 12a), not protruding in *He. garyam* and slightly protruding in *Hi. amphibius* (Figure 12e,i). On the medial lip of the proximal trochlea, a salient non‐articular oblique crest (As3) is visible in *L. bahri* (Figure 12a), with a weak stop‐facet for the tibia. It differs from the hippopotamids (Figure 12e,i), which present an elongated tibial stop‐facet and a weak to absent oblique non‐articular crest.

In hippopotamids (Figure 12f,j), the sustentacular facet and the distal trochlea (As4) are most often in contact whereas in *L. bahri* (Figure 12b), those two surfaces are separated by a non‐articular surface. In *L. bahri*, this character is sensitive to preservation issues but can be systematically observed on well‐preserved specimens.

The hippopotamids display a non‐articular stop process extending the medial lip plantarly (As5; Figure 12g,k), which is absent to very weak in *L. bahri*, although sensitive to preservation (Figure 12(c)).

The lateral fibular facet and the facet for the fibular process of the calcaneus (As6) are fused in the two hippopotamids (Figure 12(g,k)) and are clearly separated by a non‐articular surface in *L. bahri* (Figure 12c).

In distal view, the distal intracephalic fossa (As7) is limited to a narrow notch in *Hi. amphibius* (Figure 12l), which is slightly longer in *He. garyam* (Figure 12h). In *L. bahri*, this groove is much wider (Figure 12d). This character, although sensitive to preservation, is observed consistently in well‐preserved specimens.

#### 3.6.2 Calcaneum

**FIGURE 13:**
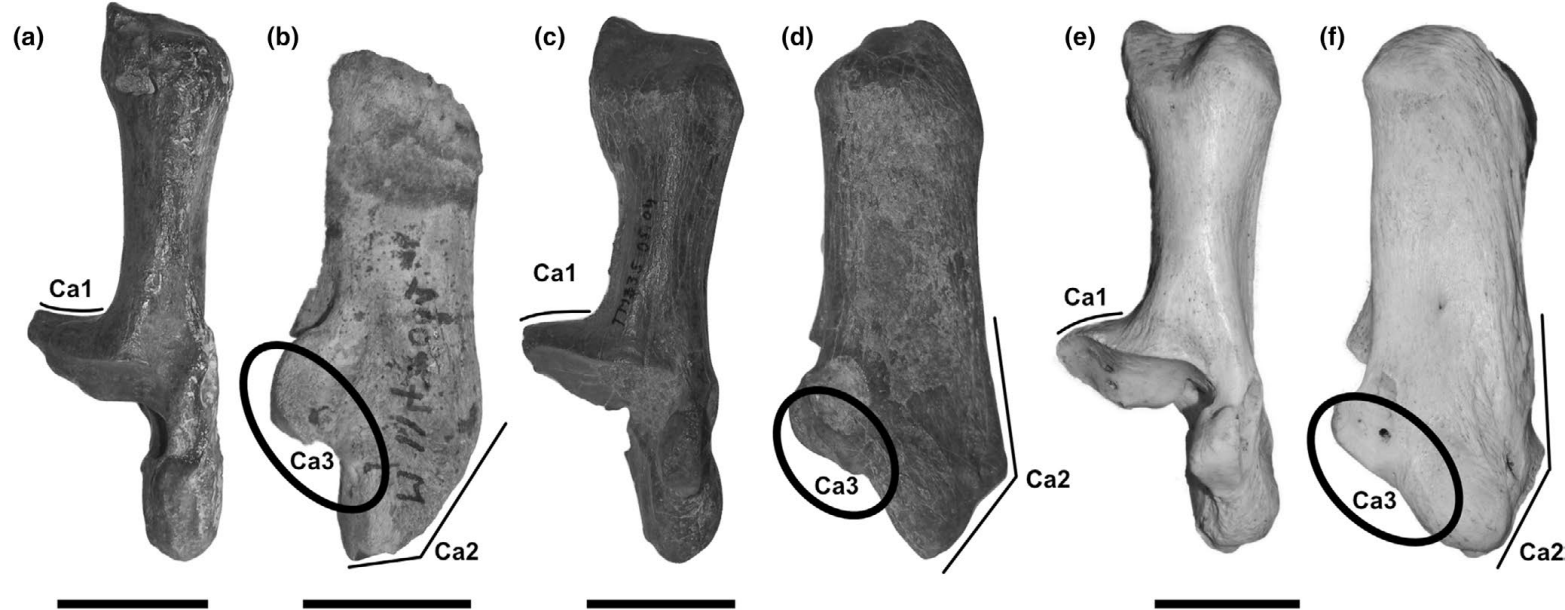
Calcanei of Hippopotamoids from TM and extant common hippopotamus. (a and b) *Libycosaurus bahri* (TM9‐00‐14, right for a; TM114‐00‐01, right for b) in cranial (a, mirrored) and lateral (b, mirrored) views. (c and d), *Hexaprotodon garyam* (TM335‐05‐04, left) in cranial (c) and lateral (d) views. (e and f) *Hippopotamus amphibius* (OST‐361, left) in cranial (e), lateral (f) views. Scale bars = 5 cm.

The projection of the sustentacular process (Ca1) is straight to concave toward the *tuber calcanei* in *L. bahri* (Figure 13a). It differs from *He. garyam* and *Hi. amphibius* (Figure 13c,e), which both display a convex mediolateral process.

In lateral view, the articular facet for the cuboid (Ca2) is more distally oriented in *L. bahri* (Figure 13b) than in *He. garyam* and *Hi. amphibius*, where it is more plantar (Figure 13d,f).

In lateral view, the notch beneath the surface for the fibular trochlea (Ca3) is more pronounced in *L. bahri* (Figure 13b) than in *He. garyam* (Figure 13d). It is even less marked in *Hi. amphibius* (Figure 13f).

In distal view, the articular surface for the sustentacular facet of the astragalus (Ca4) constitutes the entirety of the sustentacular process for the hippopotamids (Figure S5a–c).

In contrast, for *L. bahri*, this surface only constitutes approximately 75% of the process, due to the development of the *sustentaculum tali* and the presence of a developed non‐articular area (Figure 13c). The outline of the articular facet with the cuboid (Ca5) is oval to pear‐shaped in *L. bahri*, in contrast to the two hippopotamids, which present a more rectangular outline (Figure S5a–c).

#### 3.6.3 Cuboid

In lateral view, the contact surface with the calcaneum (Cb1) is rather oriented medially in *L. bahri* (Figure S6a) whereas it is oriented dorso‐proximally in the two hippopotamids (Figure S6c,e).

The distal process (Cb2) is way more pronounced in *He. garyam* and *Hi. amphibius* than in *L. bahri* (Figure S6a,c,e).

In dorsal view, there is significant variation in the compression of the cuboid bone, even taking into account the different preservation states (Cb3). *Hi. amphibius* displays a proximo‐distally compressed shape (Figure S6f; ratio calculated at 1.62). *He. garyam* and *L. bahri* (Figure S6b,d) are more proximo‐distally elongated and both have a squarer shape, with respective ratios of 1.45 and 1.40.

#### 3.6.4 Navicular

The plantar height of the bone (Nv1) is greater than the dorsal height in *L. bahri* (Figure S6g), whereas size differences are less marked in the hippopotamids (Figure S6i,k).

The articular facet with the astragalus (Nv2) is shallow in *L. bahri* (Figure S6g) in contrast with the deep facet of *He. garyam* and *Hi. amphibius* (Figure S6i,k). This is linked to the asymmetric development of the distal articular surface of the astragalus in the two hippopotamids, where the lateral one is deeper. This asymmetry is less marked in most specimens of *L. bahri*.

The plantar contact facet with the cuboid (Nv3) is proportionally larger and oriented more distally in *He. garyam* (Figure S6j), whereas it is smaller and oriented medially in *L. bahri* and *Hi. amphibius* (Figure S6h,l).

### 3.7 General comparative description: Metapodials

The three hippopotamoids are tetradactyl. It is worth noting that some Eocene anthracotheres, such as *Elomeryx*, have pentadactyl forelimbs (Scott & Jepsen, 1940).

**FIGURE 14:**
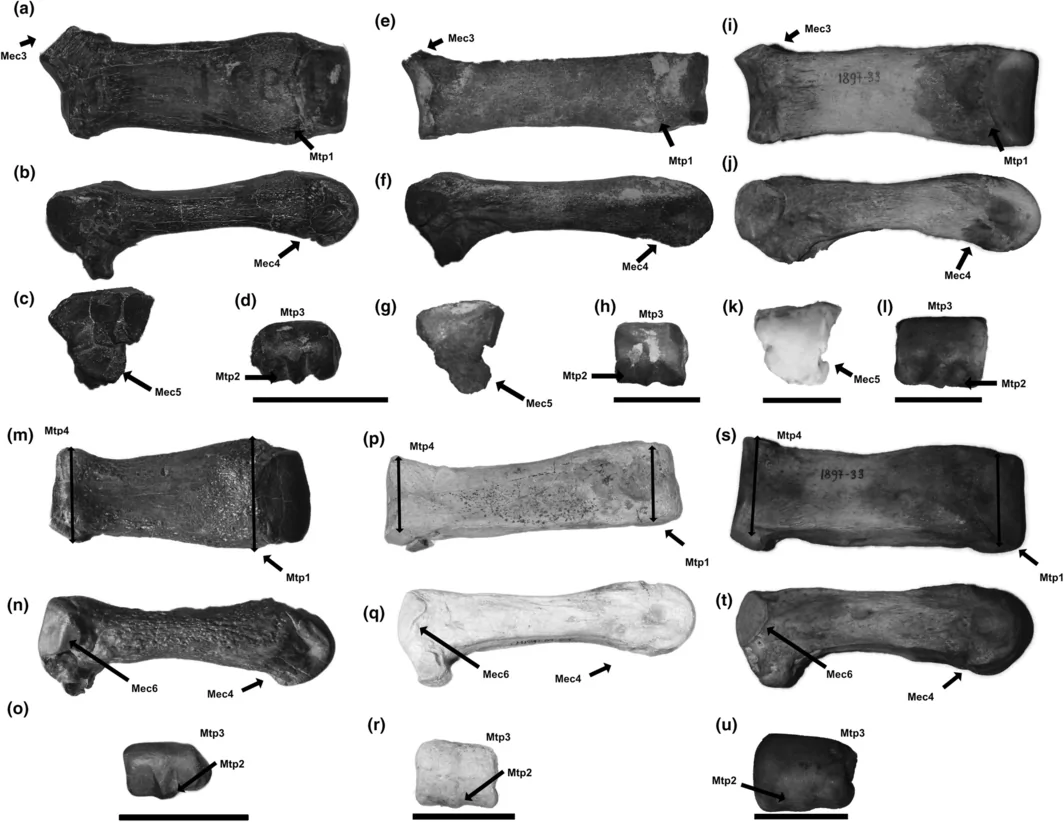
Central metacarpals of Hippopotamoids from TM and extant common hippopotamus. (a–d) MC III; *Libycosaurus bahri* (TM92‐99–002, right) in cranial (a, mirrored), lateral (b), proximal (c), and distal (d) views. (e–h) MC III; *Hexaprotodon garyam* (TM115‐00‐36, right) in cranial (e, mirrored), lateral (f), proximal (g), and distal (h) views. (i–l) MC III; *Hippopotamus amphibius* (MNHN‐1897‐33, left for i/j/l; MNHN‐1924‐134, left for k) in cranial (i), lateral (j, mirrored), proximal (k, mirrored), and distal (l) views. Scale bars = 5 cm. (m–o) MC IV; *Libycosaurus bahri* (TM123‐10‐10, left) in cranial (m), medial (n), distal (o) views. (p–r) MC IV; *Hexaprotodon garyam* (TM180‐01‐05, left) in cranial (p), medial (q), distal (r) views. (s–u) MC IV; *Hippopotamus amphibius* (MNHN‐1897‐33, left) in cranial (s), medial (t), distal (u) views. Scale bars = 5 cm.

In all metapodials, the distal articular head displays a marked keel in *L. bahri* whereas it is weaker in hippopotamids (Mtp2; Figures 14, S7–S9). The distal part of metacarpals of *L. bahri* is characterized by a notable strong swelling proximally to the articular head, regardless of the studied metapodial (Mtp1; Figures 14, S7–S9). In both hippopotamids, the metapodials tend to have straighter distal edges (Mtp1; Figures 14, S7–S9). Juveniles and certain hippopotamid individuals seem to develop a bulging closer to what is seen in *L. bahri*, but the width increases regularly in the latter while it is more localized and irregular in the former. Distally, the cross‐section of the articular surfaces with the proximal phalanges in *L. bahri* tend to be more globular and/or triangular/rhombus‐shaped, while they are generally squared to rectangular in the two hippopotamids (Mtp3; Figures 14, S7–S9). There is a marked width asymmetry between both epiphyses of the metapodials in *L. bahri*, whereas both hippopotamids generally display sub‐equal epiphyses (Mtp4; Figures 14, S7–S9).

It is important to note that the characters described above apply mostly to central metapodials, as external metapodials tend to be more variable.

#### 3.7.1 Second and fifth metacarpals

As previously mentioned, tetradactyly is confirmed here by the absence of articulation with the first metacarpal, which should be visible on the second metacarpal of all three hippopotamoids.

Proximally, the small lateral contact surface with the magnum (Mec1) is larger and more proximally oriented in *L. bahri* (Figure S7c) than in both hippopotamids. This articular surface is smaller and more lateral in *Hi. amphibius* than *He. garyam* (Figure S7g,k).

On the fifth metacarpal, it is important to note here that the median keel usually present in *L. bahri* is less clear (Figure S7o), and that this same keel is more pronounced than usual in *He. garyam* (Figure S7r). Otherwise, no characters specific to the MC V have been clearly identified.

The articular facet with the MC IV (Mec2) is wider than it is high in *He. garyam* and *Hi. amphibius* (Figure S7q) and wider than high in *L. bahri* (Figure S7n,t).

#### 3.7.2 Third and fourth metacarpals

On the third metacarpal, in cranial view, the contact surface with the unciform (Mec3) is larger and more dorsally oriented in *L. bahri* (Figure 14a) than in the two hippopotamids, where it is more lateral (Figure 14e,i). This surface is here again larger in *Hi. amphibius* than in *He. garyam*.

In lateral view, the palmar end of the articular head (Mec4) forms a beak in *L. bahri* (Figure 14b) whereas the transition between the head and the diaphysis is smooth in hippopotamids (Figure 14f,j).

The palmar process of the proximal epiphysis (Mec5) is medio‐laterally thinner and longer in both fossil forms (Figure 14c,g). In contrast, it appears square and more robust in *Hi. amphibius*, with less pronounced palmar extension (Figure 14k).

On the fourth metacarpal, *L. bahri* exhibits an additional lateral small articular facet (Figure 14n) for the MCIII (Mec6) that the two hippopotamids do not display (Figure 14q,t).

As in the MC III, the transition between the distal articular surface and the palmar side of the bone (Mec4) is beak‐shaped in *L. bahri* (Figure 14n) and is smooth and linear in hippopotamids (Figure 14q,t).

#### 3.7.3 Second and fifth metatarsals

There is a marked difference between the proximal and distal widths (Met1) of the second metatarsal of *L. bahri* and *He. garyam* (Figure S8a,d) whereas *Hi. amphibius* displays more sub‐equal widths (Figure S8g).

As for the fifth metatarsal, the surface contacting the MT IV (Met2) is flat and entirely on the medial side in *Hi. amphibius* and *He. garyam* (Figure S8m,p) whereas it is irregular and more dorsally oriented in *L. bahri* (Figure S8j). This surface is thus also much more visible in proximal view in *L. bahri* (Figure S8k), which is not the case in the hippopotamids (Figure S8n,q).

#### 3.7.4 Third and fourth metatarsals

In dorsal view, the proximal‐most edge of the metatarsal (Met3) is sigmoid in *L. bahri* (Figure S9a) whereas it is slightly concave in the hippopotamids (Figure S9f,k).

Proximally, the articular contact surface with the MT IV (Met4) is flat to convex in *Hi. amphibius* and *He. garyam* (Figure S9g,l) and markedly concave in *L. bahri* (Figure S9b).

In plantar view, *L. bahri* (Figure S9b) displays a strong depression distally (Met5), right before the distal articular surface. The two hippopotamids do not exhibit this morphology, with only a slight depression visible (Figure S9g,l).

The plantar side of the metatarsal (Met6) of the common hippopotamus is straight distally like in *He. garyam* (Figure S9h,m), whereas the entire metatarsal is curved in *L. bahri* (Figure S9c).

In proximal view, the development of the articular surface (Met7) is stronger laterally in *L. bahri* (Figure S9d) whereas it is stronger on the medial side in the hippopotamids (Figure S9i,n).

*L. bahri* also displays on this view a non‐articular surface on the dorsal‐most point of the metatarsal (Met8, Figure S9d). This non‐articular surface is absent to very weak in *He. garyam* and *Hi. amphibius* (Figure S9i,n).

On the fourth metatarsal, in plantar view, there also is a large, marked fossa (Met5) in *L. bahri* (Figure S9q), that is comparatively smaller in *He. garyam* (Figure S9v) to weak in *Hi. amphibius* (Figure 9a’).

Proximally, in *L. bahri*, the medial articular surface contacting the third metatarsal (Met9) displays a spherical configuration (Figure S9r), in agreement with the opposite contact surface described on the MT III (Met4). This surface is flat to convex in the two hippopotamids, following their MT III counterpart (Figure S9w,b').

Likewise, the contact facet with the fifth metatarsal (Met10) is more rounded and dorsally oriented in *L. bahri* (Figure S9s). In contrast, in *He. garyam* and *Hi. amphibius*, this articular surface is more rectangular and well defined laterally (Figure S9x,c').

In proximal view, *L. bahri* displays again a non‐articular surface on the dorsal‐most point of the metatarsal (Figure S9t ‐ Met8). This non‐articular surface is absent to very weak in *He. garyam* and *Hi. amphibius* (Figure S9y,d').

### 3.8 Body mass estimations

Mean body mass estimations using long bones (derived from Scott, 1983) and astragalus (derived from Martinez & Sudre, 1995) are detailed in Supplementary Material III. The detailed body mass estimations per specimen are detailed in Supplementary Material II in the corresponding tabs. For the astragalus, the weight range of *He. garyam* (Min: 994 kg, Max: 2126 kg; Mean = 1504.73 kg ± 240.04), is similar to *Hi. amphibius* (Min: 1064 kg, Max: 2583 kg; Mean = 1640.27 kg ± 662.10) although slightly more gracile. *L. bahri* is on average 300 kg lighter than both hippopotamids (Min: 700 kg, Max: 1781 kg; Mean = 1243.48 kg ± 312.62). The estimates calculated with the long bones yielded different results than the estimations using the astragalus. There were also varying results across the measurements used. Some measurements were coherent (femur length, humerus length and tibial plateau transverse width) with what is known about the weight range of the extant hippopotamus (Kingdon, 2014) and some yielded abnormal results (ulnar length, radial length and transverse width of proximal radius; see Supplementary Material III for discussion).

## 4 DISCUSSION

### 4.1 Postcranial anatomy amongst artiodactyls and between hippopotamoids

This study is the first to provide a detailed description and comparison of the postcranial anatomy of the TM hippopotamoids, offering interesting evidence to discuss the relations of the Hippopotamoidea within artiodactyls (e.g., Boisserie et al., 2005; Colbert, 1935; Gentry & Hooker, 1988; Lihoreau et al., 2014; Matthew, 1934; Pearson, 1927; Pickford, 1983, 2008).

It highlights characters (summarized in Supplementary Material IV) specific to the Hippopotamoidea that would need to be investigated across the superfamily. Firstly, the tetradactyl conformation of hippopotamoids is fully functional, with four weight‐bearing digits, which is not the case in most artiodactyls. Secondly, the autopod is compressed dorso‐ventrally, mainly because of the weight of the taxa studied. On the forelimb, the characteristic palmar processes of the carpals of the hippopotamoids are absent in most other artiodactyls. When present, these processes are very weakly developed, suggesting an increased digital mobility in organisms that evolved to be adapted to prevent splaying on soft, muddy terrains (Fisher et al., 2007). The metapodials tend to be symmetrical on their distal part (Mtp2), much more than in other artiodactyls that display non‐fused metapodials with a distal keel such as suids (Supplementary Material V).

More importantly, there is a clear absence of fovea capitis on the femoral head of the hippopotamoids. This is due to the absence of the *ligamentum teres femoris*, which has been linked to an increase in mobility of the hindlimb in primates (Muchlinski et al., 2018; O'Donnell et al., 2014) and could thus serve similar functions in hippopotamoids, despite clearly distinct general morphology between primates and hippopotamoids.

Furthermore, the absence of acetabular notch in all hippopotamoids (Figure S2 ‐ Pv1; Andrews, 1906; Ducrocq, 1999; Schmidt, 1913) is consistent with this hypothesis. This is because the *ligamentum teres femoris* usually passes through this notch; therefore, its absence allows greater freedom of movement of the hindlimb than in most artiodactyls, especially laterally.

### 4.2 Head posture and skull weight bearing

Complete skulls have been documented for *L. bahri* but not yet for *He. garyam*. Same goes for the cervical region of the axial skeleton. This makes it difficult to compare the reconstructed head postures and behaviors of the three hippopotamoids.

Extant male hippopotamuses engage in combat by clashing jaws and using the mandible and neck exhibiting behaviors akin to those observed in antler fights to establish dominance (Eltringham, 1999; Kingdon, 2014). Given their comparable morphologies, it is pertinent to inquire whether *Hex. garyam* exhibited analogous forms of intraspecific competition. Did *L. bahri* also employ jaw‐to‐jaw clashing or did they adopt other behaviors, as their different postcranial anatomy could demonstrate? (Table 2).

**TABLE 2:** Morphofunctional synthesis of characters based on this study and Fisher et al. (2007, 2010), Boisserie et al. (2005) and Lihoreau et al. (2014).

	Intraspecific competition	Mobility/locomotion	Interaction with environment
*Libycosaurus bahri*	Deep intervertebral connections (At5, Ax3) Massive neck musculature, less so than both hippopotamids (Cv1/2, At3/7, Ax2) Less powerful neck flexors (Sp2/4) Increased cranial roof thickness and wounds => Powerful less mobile neck, resistance to high anteroposterior constraints Frontal headbutting behaviors?	Elongated scapula, caudally projected humeral head (Hm7) Markedly curved humerus (Hm1), lighter and differently oriented deltoid tuberosity (Hm2) Smaller, less marked tuberosities and muscular insertions (Ru5, Fm1) Shorter femoral neck (Fm9/Fm10) More developed hindlimb (Fm4/Fm5) Relatively shorter zeugopod than stylopod => More use of the hindlimb, less propulsive force More parasagittal movements than lateral and rotational ones	Large asymmetrical metapodials (Mtp1) Locking osteological mechanisms: less mobiliy of both ankles and wrists (Tb8, As1/As2/As3, Ca2, Cb1, Mec1/Mec2/Mec6, Ru6) Digit flexors differently developed (Fm6) => Use of the autopods as stiff paddles to swim (swimming movements being coherent with the parasagittal mobility cited previously). Possible webbed autopods?
*Hexaprotodon garyam*	Shallower vertebral connections (At5, Ax3) Stronger neck musculature than in *L. bahri* (At3/7) Powerful neck flexors (Sp2/4) Large incisors and canines for fighting => Powerful mobile neck, resistance to lateral movements Jaw to jaw clashing like in extant hippopotamuses?	Strong forelimb with powerful muscles, for propulsion and stability (Sp1/Sp4, Hm2/Hm3/Hm6/Hm10, Ru3/Ru5) More laterally and rotationally mobile limbs (Hm7, Fm3/Fm9/Fm10) Limbs segments are more sub‐equal in lengths => More forelimb reliant locomotion, adaptation to propulsion, power and stability. Higher mobility laterally and rotationally Adaptation to steep riverbanks and energetic environments with high constraints? Still to a lesser degree than *Hippopotamus amphibius*?	More symmetrical metapodials (Mtp1) More mobility of both ankles and wrists (As1/As3/As6, Ca2, Cb1, Mec2/Mec3/Mec6/Met3/Met9) Digit flexors differently developed (Fm6, Hm8/Hm9, Ru6) => High mobility of the autopod, especially between digits High mobility of the wrist and ankle, playing a role in weight support but also stability
*Hippopotamus amphibius*	Shallower vertebral connections (At5, Ax3) Stronger neck musculature than in *L. bahri* (At3/7) Powerful neck flexors (Sp2/4) Large incisors and canines for fighting => Powerful mobile neck, resistance to lateral movements	Strong forelimb with powerful muscles, for propulsion and stability (Sp1/Sp4, Hm2/Hm3/Hm6/Hm10, Ru3/Ru5) More laterally and rotationally mobile limbs (Hm7, Fm3/Fm9/Fm10) Limbs segments are more sub‐equal in lengths => More forelimb reliant locomotion, adaptation to propulsion, power and stability. Higher mobility laterally and rotationally Adaptation to steep riverbanks and energetic environments with high constraints?	More symmetrical metapodials (Mtp1) More mobility of both ankles and wrists (As1/As3/As6, Ca2, Cb1, Mec2/Mec3/Mec6/Met3/Met9) Digit flexors differently developed (Fm6, Hm8/Hm9, Ru6) => High mobility of the autopod, especially between digits High mobility of the wrist and ankle, playing a role in weight support but also stability

Cranial adaptations consistent with headbutting behaviors in *L. bahri* have already been mentioned such as the increased cranial roof thickness (Lihoreau et al., 2014). Furthermore, healed wounds on the skull of *L. bahri* (Lihoreau et al., 2014) could also point towards this form of intraspecific competition. The canines and incisors of *L. bahri* are much smaller than in extant hippopotamuses, which lessens but however does not rule out the possibility that these wounds have been alternatively inflicted by strong bites.

The massive fused mandibular symphysis and enlarged, ever‐growing incisors at the front of the mandible of this merycopotamin suggest a weight distribution much more front‐heavy than in other mammals (Lihoreau et al., 2014), but similar to that of hippopotamids (Boisserie, 2007). Complementing this hypothesis, *L. bahri* seems to display a massive neck musculature as in the extant *Hi. amphibius* based on the neck vertebrae morphology (Cv1, 2; At3; Ax2). The relatively high weight and size of the head of these taxa implies the presence of ligaments and muscles allowing the animal to keep a quite stereotypical neck resting posture. The development of these muscles, such as the *M. omo‐transversarius* and *Mm. serrati ventral*, can clearly be seen in the development of the processes of the two first cervical vertebrae (Figure 2g,h ‐ At3, At7; Figure 3a ‐ Ax1) and is linked to neck stability, mobility, and power.

The similarities between the cervical vertebrae of both hippopotamids suggest that *He. garyam* closely resembled the common hippopotamus in terms of cranial and neck mobility and usage. A jaw‐to‐jaw clash would necessitate an increased mediolateral stability to avoid offsetting the contact point of the mandibles with the addition of strong neck muscles to support the strain on the neck. The neck musculature would therefore be more important in both hippopotamids than it was in the merycopotamin. A frontal headbutting would likely require a form of stability that was more anteroposterior or dorso‐ventral than medio‐lateral. This is consistent with the size and depth of the cranial articular surfaces of the atlas of *L. bahri* (Figure 2b ‐ At5), which are deeper, higher, and represent overall a tighter fit and contact with the skull than in both hippopotamids in which the surfaces tend to be shallower (Figure 2e,h ‐ At5).

The atlases of the two fossil hippopotamoids are nonetheless flatter, thinner, and less robust than in the common hippopotamus. This suggests that even if the forces involved in stabilizing the neck and the bearing of the head load were very strong in all three taxa, the extant hippopotamus possesses a stronger set of muscles to deal with these constraints (Arnold, 2021). This can also be seen in the strong ventral processes present in the third to seventh cervical vertebrae of *Hi. amphibius*, which are only weakly developed to almost absent in *L. bahri*. This suggests that the axial skeleton of the extant hippopotamus is more derived, powerful, and potentially specialized for the constraints implied by a jaw‐to‐jaw clashing.

On the axis, the angle between the parasagittal plane and the prezygapophyses (Figure 3b,f,j ‐ Ax3) is greater in *L. bahri* than in *He. garyam* and *Hi. amphibius*. Inversely with the atlas, the thickness of the spinous arch and both posterior arches are significantly greater in *L. bahri* than in *He. garyam* and *Hi. amphibius*. This could suggest that the bulk of the stability of the neck of *Libycosaurus* was governed to a greater extent by vertebral interactions and less by strong muscles, in contrast with the opposite condition of the two hippopotamids (Figure 2d,g,e,h ‐ At3, 7). This lends further support to a lesser mediolateral “flexibility”, that is that vertebral morphology is not only linked to head size and weight but also to its usage and the constraints that go with it.

The rest of the postcranial skeleton, mainly the forelimb and pectoral girdle also play a significant role in neck usage, posture and head weight bearing. Indeed, many muscles such as the *M. omo‐transversarius* link the cervical vertebrae with part of the pectoral girdle (Fisher et al., 2007). For instance, the *M. omo‐transversarius* originates from the alar processes of the atlas and the articular processes of the axis and reaches the scapular spine and acromion process. It plays a role in many key functions such as advancing the forelimb but more importantly flexing the neck medio‐laterally. Character‐wise, we can observe the very strong development of the spine and acromion process in *Hi. amphibius*, and less so in *L. bahri* (Figure 5a,e ‐ Sp2). This indicates that the neck of *L. bahri*, even if less mobile than at least *Hi. amphibius*, still keeps moderate mobility, potentially for feeding behaviors (Eltringham, 1999; Kingdon, 2014). It is important to note that other muscles attached to the scapula, such as the *Mm. serrati ventrale*, which inserts on its caudal angle, also have a significant impact on head posture and neck mobility. The cervical part of this muscle complex is massively developed in *Choeropsis liberiensis* and *Hi. amphibius* and takes its origin on the alar processes of the atlas (Fisher et al., 2007). It also inserts at multiple places along the dorsal border of the scapula (Fisher et al., 2007) and along the first to ninth rib. This set of muscles is responsible for neck flexion medio‐laterally and dorso‐ventrally. Thus, the thick dorsal border of the scapula (Figure 5b,f ‐ Sp4) of *Hi. amphibius* contrasts with the more tenuous border of *L. bahri* (even when damaged). This suggests that both taxa displayed powerful neck muscles but supplements the fact that *Hi. amphibius* has stronger complexes, probably due to a heavier head and greater constraints when fighting. The *Mm. rhomboidei* is also a muscle complex whose cervical part flexes the neck (Fisher et al., 2007). As previously cited, both muscle complexes originate in the lumbar region but attach almost to the entirety of the dorsal border of the scapula. The dorsal border and caudal angle of the scapula of *Hi. amphibius* are stronger and larger than those of *L. bahri*. This provides further evidence to the hypothesis that the neck of the common hippopotamus is more mobile and stronger, in contrast to the more column‐like neck of the merycopotamin. It is important to note that previous studies showed similar column‐like straight neck postures in anthracotheres (Geais, 1934; Pickford, 2022; Scott & Jepsen, 1940).

### 4.3 Posture, locomotion, and mobility across different environments

Some possible semi‐aquatic adaptations on the musculoskeletal limb morphology of extant hippopotamuses have been previously suggested, notably muscular insertions on the hindlimb and forelimb metapodials (Fisher et al., 2007, 2010). It is important to study these features across multiple genera in the fossil record and throughout the evolutionary history of Hippopotamoidea to assess if these adaptations are unique to extant hippopotamids, or if they can be found across the superfamily. Do the similar limbs of both hippopotamids show that *He. garyam* behaved in a similar way to *Hi. amphibius*? Since both TM hippopotamoids co‐occurred in similar environments, did *L. bahri* employ a different locomotion type, and thus exploit the aquatic environments of TM differently (Table 2)?

The scapula of *L. bahri* is more elongated than in the extant common hippopotamus and has a shallower glenoid cavity. Both of these features suggest a potentially greater parasagittal amplitude of the forelimb (Matsuo et al., 2019) in *Libycosaurus*.

Also, the caudal projection and the more anteroposteriorly elongated humeral head (Figure 6a,e,i ‐ Hm7; Figure S1a–c ‐ Sp5) of *L. bahri* seem to allow greater anteroposterior movement. However, the humeral head of both hippopotamids seems to allow greater control of the lateral and medial rotation of0 the forelimb.

The humerus of *Libycosaurus* is markedly curved at the deltoid tuberosity (Figure 6a,e,i ‐ Hm1) compared to both hippopotamids, which display a more column‐like humerus. In mammals, humerus curvature is highly variable, reflecting a more complex cause or function at the origin of this curvature (Bertram & Biewener, 1992; Henderson et al., 2017; Milne, 2016). In *L. bahri*, the deltoid tuberosity is cranially shifted compared to in both *Hi. amphibius* and *He. garyam* (Figure 6a,e,i ‐ Hm2). The three muscles (*M. deltoideus, M. brachiocephalicus, M. pectoralis superficialis*) attached to this more cranial tuberosity are thus suggestive of a specialization toward more parasagittal movement of the forelimb (Fisher et al., 2007). Both hippopotamids have noticeable insertion surfaces on their deltoid tuberosity while still displaying weak to almost absent curvature on their humeri. This indicates that the peculiar curvature of the humerus of *L. bahri* is of a different origin and is not caused by the action of the muscle complexes mentioned previously.

The humeral head of both hippopotamids does not exhibit a marked distal projection (Figure 6a,e,i ‐ Hm7), while *L. bahri* displays a strong distal projection of the articular surface. This demonstrates a difference in the connection between the scapula and humerus (also seen in the morphology of the glenoid cavity), where *L. bahri* probably had a less vertical configuration of the proximal part of the forelimb, in contrast to the almost entirely vertical scapula of the hippopotamids. This supplements the previous hypothetical wider range of anteroposterior movement of *Libycosaurus*, trading off the more powerful muscles, stability and weight support of the hippopotamids for more mobility. This mobility could potentially be used for drag‐based swimming, as in paddling (Fish, 1996). *Hi. amphibius* is capable of paddling but is too susceptible to drag and constraints in water, mainly due to its massive weight. This prevents it from swimming like most semi‐aquatic quadrupedal mammals (Coughlin & Fish, 2009; Fish, 1994). The common hippopotamus thus relies on the increased buoyancy to adopt a locomotion analogous to movement in a microgravity environment (Coughlin & Fish, 2009). The locomotion of *L. bahri* could be different from the former, notably due to being lighter but also having limbs better adapted to anteroposterior paddling. As *C. liberiensis* is much lighter (Eltringham, 1999; Kingdon, 2014), and yet still displays proportionally similar muscle development (Fisher et al., 2007), it is important to note that body mass might have less impact on bone morphology and that a phylogenetic signal or locomotor signal might play a more important role in shaping the postcranial skeleton. This has also been shown in other fossil hippopotamids such as the endemic Cypriot pygmy hippopotamus *Phanourios minor* (Georgitsis, Liakopoulou, & Theodorou, 2022; Georgitsis, Liakopoulou, Theodorou, & Tsiolakis, 2022).

The medial side of the diaphysis of the humerus of both hippopotamids shows a clear marked insertion for the *M. teres major* (also visible in the scapular caudal border discussed previously) and *M. latissimus dorsi* (Fisher et al., 2007). These muscles are involved in stabilizing the trunk, but above all in propelling it forward (Fisher et al., 2007). This marked muscular insertion zone (Foster et al., 2014) is weaker to almost absent in *L. bahri*, possibly indicating that the merycopotamin did not need as much propulsion strength as *Hi. amphibius* and *He. garyam*. In hypothetical headbutting confrontations, propulsion strength also could have had functional importance, thus also explaining the need for powerful muscles in *L. bahri*, even if less used for locomotion.

In both *Hi. amphibius* and *He. garyam*, the olecranon process is markedly curved laterally (Figure 7b,e,h ‐ Ru5). However, no such curvature is visible in *L. bahri*, which indicates that the triceps is more developed in the hippopotamids. The main role of this muscle complex is to stabilize the elbow and shoulder joints when standing, which is coherent with the average superior body mass of the hippopotamids. The triceps is the muscle that mainly directs the morphology of the ulnar proximal end. The three heads of the triceps (*mediale*, *longum*, and *laterale*) allow for better stability of the elbow joint when standing but also serve in extending this joint (Fisher et al., 2007). The curvature of the ulna of the two hippopotamids indicates a more powerful extension of the elbow joint and stability of the shoulder joint compared to *L. bahri* (Henderson et al., 2017). Overall, all former characters supply the hypothesis that the hippopotamids possessed more propulsion force than the merycopotamin, possibly linked to an adaptation to climbing steep inclines in muddy riverbanks (Fisher et al., 2007, 2010). More so, the need for support and stability would be greater in *He. garyam* and *Hi. amphibius*, due to the increased muscle development and thus greater constraints (Bels & Russell, 2023; Boisserie et al., 2005; Fisher et al., 2007; Lihoreau et al., 2014).

The hippopotamids possess a strong caudal projection on the humerus medial epicondyle, whereas it is projected medio‐caudally in *L. bahri*. In addition to enabling movement of the digits (see below), the muscles inserting into this area, namely the *M. flexor carpi radialis* and *flexor carpi ulnaris* (Fisher et al., 2007), play a pivotal role in the flexion and extension of the elbow joint. This change in the projection orientation could also suggest a difference in the forearm mobility although it seems more closely linked to the mobility of the digits (Fisher et al., 2007). The articulation with the radioulna also differs between *L. bahri*, displaying a dominant capitulum compared to the trochlea, and the hippopotamids, displaying the opposite condition (Figure 6d,h,l ‐ Hm10). This pattern is also expressed on the proximal part of the radioulna (Ru4) and could indicate a change in the “resting” position of the forelimb in *Libycosaurus* compared to both hippopotamids. Indeed, the hippopotamids seem to have a more inwards resting position of the forelimb, supplemented by the medially curved olecranon, potentially allowing for more support and stability of the shoulder and elbow joints. *L. bahri* in opposition would have a more outward resting forelimb, or generally more perpendicular to the ground. Despite these differences, all three hippopotamoids seem to adopt similar postures as they share relatively close femoral anteversion. This kind of torsion has biomechanical advantages probably linked to the reduction of the strains and forces applied to the bone during classical locomotion (Tayton, 2007).

*L. bahri* has a shorter and more vertical femoral neck (Figure 10c,f,i ‐ Fm9, 10). The hindlimb of *Libycosaurus* is thus probably positioned closer to its body. Ruminants usually have a hindlimb positioned closer to their body and are probably linked to an increased usage of parasagittal movements. The *M. gluteus medius* inserted on the greater trochanter of the femur plays a role in propelling the trunk forward and in rotating weakly the femur medio‐laterally (Fisher et al., 2010). Both hippopotamids show greater muscular insertions on the trochanter (Figure 10a,d,g ‐ Fm1), meaning that the *M. gluteus medius* is stronger in these taxa and that the abduction power is thus greater. Additionally, the attachment for the *M. iliopsoas*, which flexes the hip joint and laterally rotates the femur (Fisher et al., 2007), on the lesser trochanter (Figure 10c,f,i ‐ Fm7), is clearly more developed in *L. bahri* than in *Hi. amphibius* (Fisher et al., 2010) and *He. garyam*. As this muscle complex flexes the hip joint, this provides more evidence of the increased mobility of the hindlimb, especially parasagittal, in *Libycosaurus* and is probably linked to a different ecological niche (Boisserie et al., 2005; Lihoreau et al., 2014). The development of the hindlimb of the merycopotamin is also expressed on the distal part of the diaphysis of the femur, where strong muscular and ligamentous insertions are visible (Figure 10a,d,g ‐ Fm5), more precisely of the *M. gastrocnemius* and the medial collateral ligament. They play a role in flexing the stifle (knee) joint and extending the hock (ankle) joint (Fisher et al., 2010). The difference between the three hippopotamoids could suggest that *L. bahri* had a hindlimb that was more adapted to ground locomotion and parasagittal movements, and that it employed most of the time a locomotion differing from that of both hippopotamids, with limbs more adapted for powerful propulsion and stability (Lihoreau et al., 2014). The increased usage of the hindlimb in the form of parasagittal movement could mean two possible locomotion types: a running locomotion usually seen in quadrupedal artiodactyls and a paddle‐like swimmer. The running hypothesis is not favored by the numerous locking mechanisms visible in the limbs of *L. bahri* (Figure 7c,f,i ‐ Ru6; Figure 11d,h,l ‐ Tb8; Figure 12a,e,i ‐ As2, 3). These articular and non‐articular surfaces indicate less mobility of the wrist and ankle, which is a condition opposed to the highly parasagitally mobile autopods of most running artiodactyls (Belyaev et al., 2025). In highly specialized cursorial artiodactyls, running serves a different purpose, specifically as a means of survival against predators, and thus being adapted to this locomotion type is capital for these organisms and their evolutionary success. It is important to keep in mind the fact that extant hippopotamuses are still capable of running at high speeds (30 km/h; Coughlin & Fish, 2009; Hutchinson & Pringle, 2024), but that they do not spend most of their time employing this locomotion type, mainly because predation on adult individuals remains very rare (Estes, 1992; Schaller, 2009). It is thus possible that *L. bahri* was capable of running, but that it also used its limbs to paddle in shallow freshwater bodies.

In terms of intralimb proportions between humerus and radioulna, the mean ratio (humerus over radius) for all specimens of *Hi. amphibius* and *He. garyam* stands at around 1.28, whereas the mean ratio for *L. bahri* is around 1.50. The majority of heavy quadrupeds tend to fall within these ranges (>1; Mallet, 2020), but the merycopotamin has a relatively shorter antebrachium (zeugopod) compared to the stylopod than the two hippopotamids, where the segments are more sub‐equal in length.

It has been suggested that intra‐hindlimb proportions do not vary as much as in the forelimb, probably because of the major support role that the hindlimb plays (Schmidt & Fischer, 2009). In this study, we obtained the following ratios for femur length over tibial length of 1.38 and 1.40 for *Hip. amphibius* and *He. Garyam*, respectively, and of 1.32 for *L. bahri*. This indicates that, compared to the hippopotamids, the merycopotamin possesses a hindlimb with a relatively longer tibia.

The locomotor behaviors of the studied hippopotamid are found to rely more on the forelimbs than in *Libycosaurus*, as shown by the musculoskeletal adaptations that are observable on their stylopod and zeugopod. This increased usage of the forelimb is linked to the necessity for stability of the joints and movement power, which could be related to the need for them to climb riverbanks (Table 2).

*L. bahri* exhibits an inverse trend, characterized by a more developed hindlimb, which could be associated with a different locomotion (running and possibly paddle‐swimming; Boisserie et al., 2005; Lihoreau et al., 2014, 2021) (Table 2). Other hippopotamids from the Pleistocene, notably around the Mediterranean area, have also been hypothesized as having a different locomotion compared to the extant hippopotamuses. *Phanourios minor*, *Hippopotamus pentlandi*, or *Hippopotamus creutzburgi* seemed to favor more grounded locomotion, as they inhabited areas with more uneven and mountainous terrain (Boekschoten & Sondaar, 1966; Georgitsis, Liakopoulou, & Theodorou, 2022; Georgitsis, Liakopoulou, Theodorou, & Tsiolakis, 2022).

Extant common hippopotamuses display naturally flexed limbs, as opposed to the more pillar‐like organization of heavier, and more importantly true graviportal mammals such as elephantids (Belyaev et al., 2025). The graviportal nature of the common hippopotamus has been the subject of discussion in literature (Carrano, 1999; Hildebrand, 1974; Mallet et al., 2019), with alternating classification as mediportal animals with rhinoceroses (Bader et al., 2023; Mallet, 2020), and real graviportal animals (Gregory, 1912; Osborn, 1929). However, body mass constitutes a major part of the constraints applied on the bones when the animal is moving on land (Houssaye et al., 2021). Mobility and locomotor adaptations aside, these three hippopotamoids are heavy quadruped mammals. Usually classified into the ‘megaherbivore’ ecological group, for example, (Owen‐Smith, 1988), keeping this condition in mind is important when analyzing bone morphology (Supplementary Material III). The significantly lighter body mass of *Libycosaurus* has a clear impact on the morphology of the long bones and of the more strongly involved weight‐bearing bones such as the astragali, cuboid, and distal row of the carpal region (Bertram & Biewener, 1992; Christiansen, 2002; Mallet, 2020). The long bones of *Hi. amphibius* and *He. garyam* are thus more columnar and robust on average than *L. bahri*, which tends to have relatively longer and slenderer bones with a more “ruminant‐like” bone architecture (Figure 6a ‐ Hm1; Figure 11a ‐ Tb3; Figure S6b,d,f ‐ Cb3), although this varies depending on the bone considered (Table 1).

### 4.4 Substrate/autopod interaction

Nowadays, extant hippopotamids (i.e., *Hi. amphibius* and *C. liberiensis*) are the only artiodactyls that still possess four functional weight‐bearing digits (with fully functional digits II and V, as opposed to the reduced second and fifth metapodials of some suoids and cervoids). This condition has been considered as quite primitive amongst artiodactyls as it mirrors a condition known in early representatives of the group (Clifford, 2010). However, it appears that extant hippopotamuses display an array of adaptations of the autopod linked to their respective habitats (Fisher et al., 2007, 2010; Houtekamer & Sondaar, 1979). On their anterior and posterior digits, hippopotamuses present a set of flexor and extensor tendons and muscles (*M. flexor carpi radialis*, common extensors and flexors, and a complex web of *interossei* muscles) that assist in individual control of the digits, mostly to prevent splaying on soft, muddy terrains like in the humid forests where *C. liberiensis* is present, or in the African waterbodies where *Hi. amphibius* is currently living (Fisher et al., 2007, 2010) (Table 2).

The general morphological similarities between metapodials of *Hi. amphibius* and *He. garyam* could be indicative of the functional importance of digit mobility across the hippopotamid family, in association with habitats around waterbodies and muddy, soft terrains (Table 2). In *L. bahri*, almost all metapodials display a strong and visible distal enlargement or asymmetry (Figure 14; Figures S7–S9 ‐ Mtp1). This characteristic feature of the genus suggests that individual digital mobility was lower in *Libycosaurus*. The metapodials of *Libycosaurus* also display a global reduction of the proximal articulation with the carpals and tarsals. In addition to being smaller, these proximal articulations present articular facets being more cranially oriented (Figure 14—Mec1, Mec2, Mec5). This type of articulation allows for a different digit mobility with a closer and tighter carpal and tarsal joints. Entire wrist and ankle regions have already been reported in island hippopotamuses such as *Hi. pentlandi* or *Phanourios minor* (Caloi & Palombo, 1995; Georgitsis, Liakopoulou, & Theodorou, 2022; Georgitsis, Liakopoulou, Theodorou, & Tsiolakis, 2022) and interpreted in that case as regions with reduced mobility. This pattern is also observed in *L. bahri*, but it may be linked to another adaptation. Indeed, this condition is more often found in running mammals than in graviportal ones. For instance, in *L. bahri*, both the astragalus and tibia demonstrate reduced mobility in the morphology of their articular facets (Figure 12a ‐ As1, As2; Figure 11d ‐ Tb8): the astragalus displays a marked non‐articular crest (Figure 12, As2) that lacks or that is weak in the two hippopotamids. There is also a clearly developed stop‐facet with weak to absent non‐articular crest in *Hi. amphibius* and *He. garyam*, whereas *Libycosaurus* displays a salient non‐articular oblique crest with a weak stop‐facet (Figure 12, As3; Table 2). Both these characters prevent the overextension or flexion of the ankle of the merycopotamin. Moreover, the calcaneus displays a distinctly more distal orientation of the facet with the cuboid (Figure 13b ‐ Ca2), resulting in a flatter, less mobile contact compared to the hippopotamids. The same kind of surface reduction and facet orientation on the cuboid (Figure S6a,c,e ‐ Cb1) leads to a tighter and less mobile contact between both rows of the tarsal bones. *Libycosaurus* displays an additional articular stop‐facet with the scaphoid on the radioulna (Figure 7c,f,i ‐ Ru6), that limits its wrist mobility unlike in the two hippopotamids. Conversely, hippopotamids possess an independent carpal and carpo‐metacarpal joint, rendering the whole manus/wrist complex and pes/ankle more mobile (Fisher et al., 2007, 2010). Differences in epicondyle development of the femur affect the digit mobility in *Libycosaurus* unlike in hippopotamids, with a more developed medial epicondyle linked to the *M. flexor carpi radialis*, which flexes the carpal and carpo‐metacarpal joints of the first digits (Fisher et al., 2007). The characters cited previously, especially the digit mobility and the multiple locking mechanisms found on the articulations of *L. bahri*, could add evidence to the fact that the merycopotamin was capable of swimming using its limbs and autopods as stiff paddles, in contrast to both hippopotamids, which are potentially more adapted to powerful propulsion and highly mobile autopods, preventing splaying on soft ground (Fisher et al., 2007, 2010) (Table 2).

## 5 CONCLUSIONS

In this study, we describe and compare for the first time a sample of 712 postcranial remains including 575 fossil specimens from *Hexaprotodon garyam* and *Libycosaurus bahri*, from the Miocene of Northern Chad, in Toros‐Menalla. We draw morphofunctional and paleoecological hypotheses from postcranial anatomy:

We observed different adaptations in the cervical region that suggest means of intraspecific competition coherent with the literature: for *He. garyam*, the jaw‐jaw fighting hypothesis known in the living extant specimens is privileged, notably according to neck structure and musculature but also to mandibular symphysis size, shape, and rostral dentition (Boisserie et al., 2005); for *L. bahri*, the skull structure (Lihoreau et al., 2014) and the cervical vertebrae suggest a more frontal, direct head‐to‐head fighting hypothesis.

*Hexaprotodon garyam* was potentially better adapted to more energetic environments such as rivers with steep banks (Eltringham, 1999; Kingdon, 2014). This would have involved more propelling power, individual control of the digits to be able to move efficiently on those slopes but also to be able to stand and move on soft, muddy terrains. Despite these locomotor adaptations, the heavy weight of these taxa would have also required more robust non‐curved bones, which is consistent with microanatomical specializations closely resembling *Hip. amphibius* (Houssaye et al., 2021).

*Libycosaurus bahri* has already been associated with aquatic environments in close relationship with *He. garyam*, based on cranio‐dental data (Boisserie et al., 2005; Houssaye et al., 2021; Lihoreau et al., 2006; Lihoreau et al., 2014; Orliac et al., 2023), but could have adopted a different strategy that was more associated with a “classical” terrestrial locomotion in an aquatic environment. Indeed, adding to the lake‐hypothesis of Lihoreau et al. (2014), *L. bahri* seems to possess more affinities towards moving on flatter surfaces (in contrast to the steep banks for hippopotamids), such as more parasagittal locomotion ability, less mobile zeugopod‐autopod articulation (wrist and ankle), while still retaining manus and pes digital control, but to a lesser extent than in *Hip. amphibius* and *He. garyam*. The merycopotamin would have evolved in lacustrine environments with lower water depths and flatter banks (Lihoreau et al., 2014). Semi‐aquatic mammals are generally restricted to shallow freshwater bodies such as some lakes (Fish, 1996), and thus, the multiple osteological characters studied and analyzed previously could also suggest *L. bahri* as a paddle swimmer, or at least as a heavy quadrupedal artiodactyl that was better adapted to paddle‐swimming than both hippopotamids.

*Libycosaurus* was on average lighter than both hippopotamids, which would alleviate the need for thick, greatly robust bones (like in the hippopotamids) and would therefore leave space for more curvature in the long bones. More generally, this marked curvature also indicates that *L. bahri* had greater parasagittal amplitude in both limbs and thus used more of these kinds of movement in its locomotion. These movements, which are usually linked to running adaptations in quadruped terrestrial artiodactyls (Bertram & Biewener, 1992; Henderson et al., 2017), could also be the result of an animal that could swim, considering that it spent most of its time submerged in shallow freshwater bodies.

With this study, we provide a framework for the identification of postcranial bones across Hippopotamoidea and complement the data already collected on cranio‐dental material, microanatomy, intracranial anatomy, and geochemistry. This work creates the foundation for a comparative dataset to be extended in order to study the rest of the superfamily and shows the importance of postcranial anatomy in reconstructing paleoecologies, but also the relevancy of these data when considering future phylogenetical analyses that will help in the understanding of the evolutionary history of hippopotamoids.

## AUTHOR CONTRIBUTIONS

L.S., F.L., A.H., and J.‐R.B. contributed to the conception and design of the study, L.S., F.L., and J.‐R.B acquired the data, L.S., F.L., A.H., and J.‐R.B analyzed and interpreted the data, L.S. drafted the manuscript and prepared figures and/or tables. All authors reviewed, edited, and approved the final draft of the manuscript.

## Supporting information


**Data S1:** Supplementary Figures.

**Data S2:** Supplementary Tables.

**Data S3:** SuppMatI_Material_Inventory.

**Data S4:** SuppMatII_Limb_Measurements.

**Data S5:** SuppMatIV_Character_List.

**Data S6:** SuppMatV_Suid_Comparative_Description.

## Data Availability

All material is deposited in the CNRD, Chad and is available on demand. The data that support the findings of this study are available from the corresponding author upon reasonable request.
