# Supplementary material for: Postcranial anatomy of the Miocene hippopotamoids of Toros‐Menalla, Chad
Source: J Anat. 2026 Mar 19:10.1111/joa.70135. Online ahead of print. doi: 10.1111/joa.70135 (PMC13399167; doi:10.1111/joa.70135)

Figure S1. Scapula of Hippopotamoids from TM and extant common hippopotamus.

A, *Libycosaurus bahri* (TM226-08-48, right) in proximal (A) views*.* B*, Hexaprotodon garyam* (TM337-04-02, left) in proximal views (B, mirrored)*.* C*, Hippopotamus amphibius* (MNHN-1944-166, right) in proximal (C) views*.* Scale bars = 10 cm.


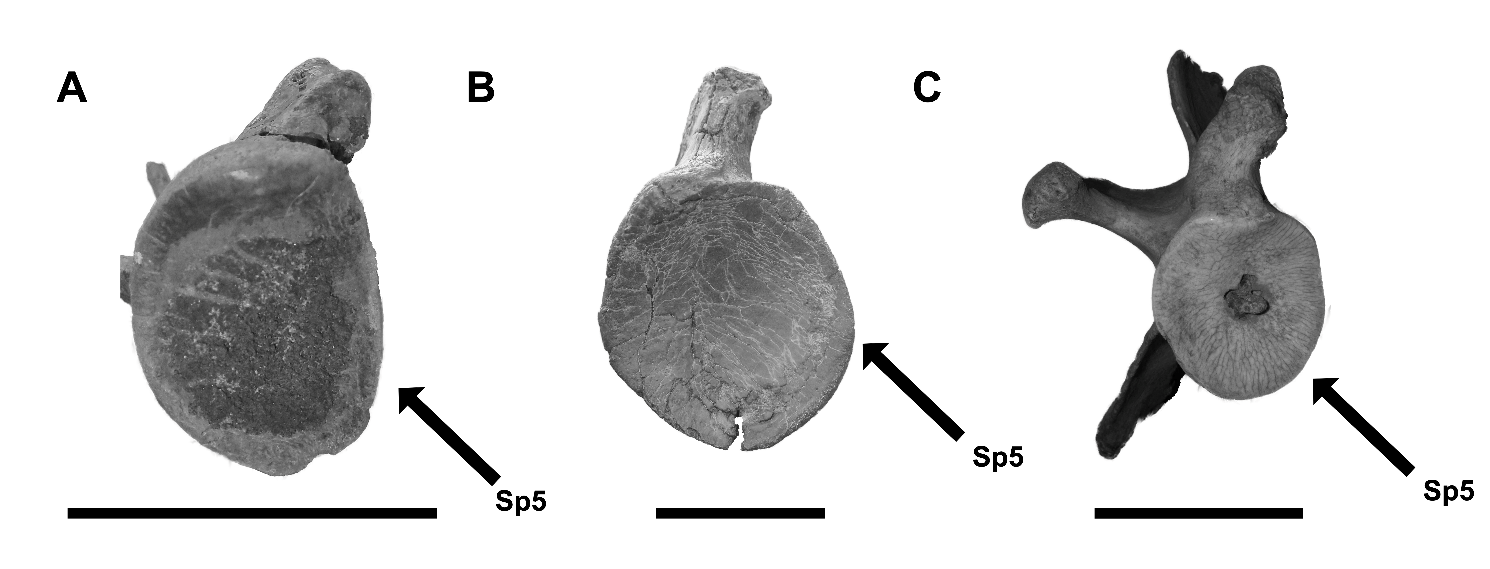


Figure S2. Pelvic bone of Hippopotamoids from TM and extant common hippopotamus.

A, *Libycosaurus bahri* (TM379-04-12b) in lateral view (A)*.* B*, Hippopotamus amphibius* (MNHN-1897-33) in lateral view (B)*.* Scale bars = 10 cm.

*
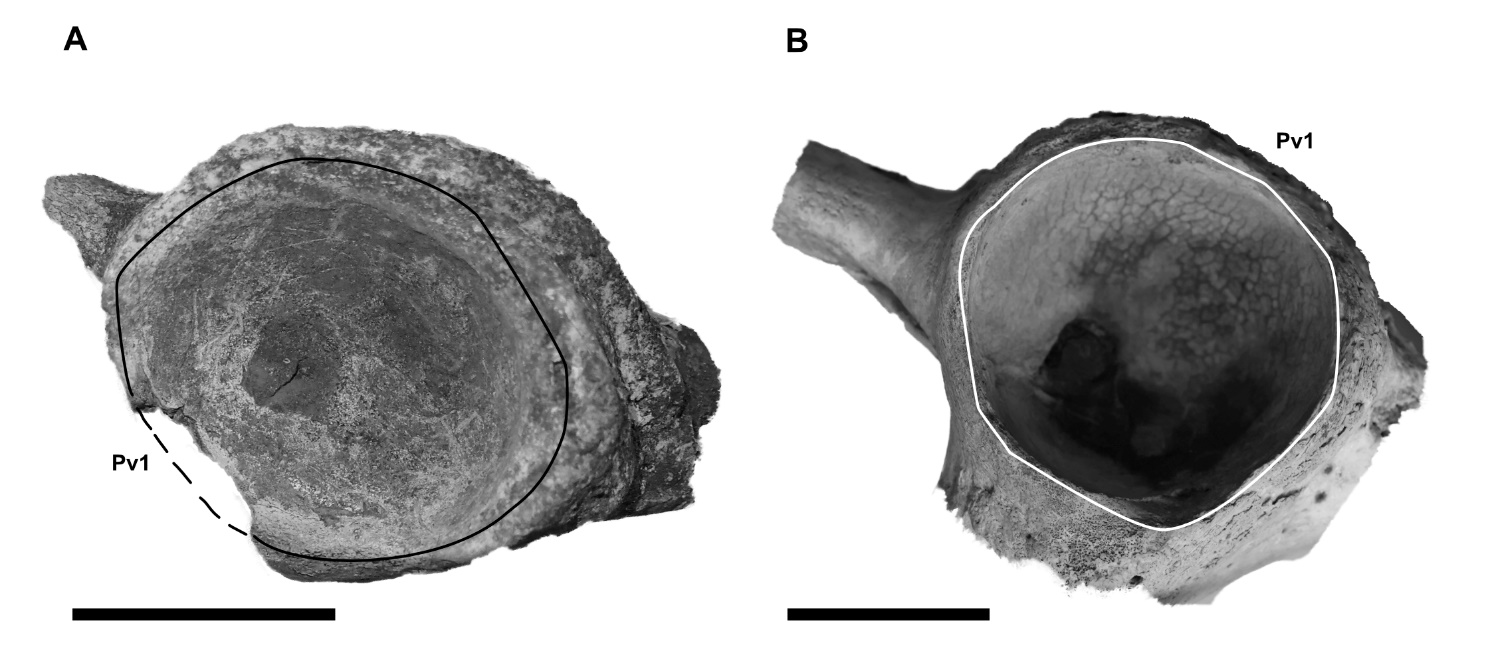
*

Figure S3. Pyramidals of Hippopotamoids from TM and extant common hippopotamus.

A*,* Pyramidal; *Hexaprotodon garyam* (TM180-01-15, left) in lateral (A) view. B*,* Pyramidal; *Hippopotamus amphibius* (30.002123, left) in lateral (B) view. Scale bars = 5 cm.


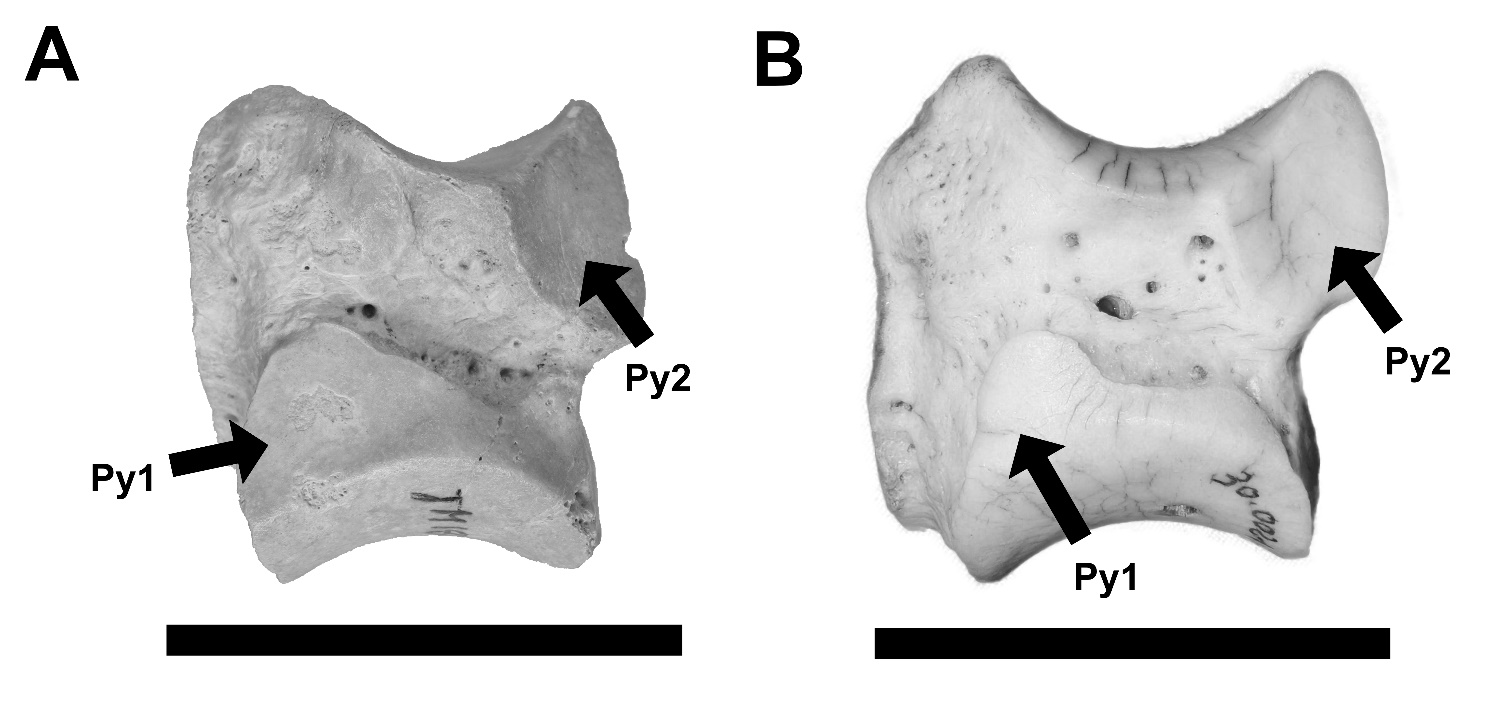


Figure S4. Patellas of Hippopotamoids from TM and extant common hippopotamus.

A*,* Patella*; Libycosaurus bahri (*TM115-XX-03*,* left, mirrored*)* in cranial (A) view*.* B, Patella*;* *Hexaprotodon garyam* (TM115-00-20, left, mirrored) in cranial (B) view*.* C, Patella*;* *Hippopotamus amphibius* (OST-360, right) in cranial (C) view*.* Scale bars = 5 cm*.*


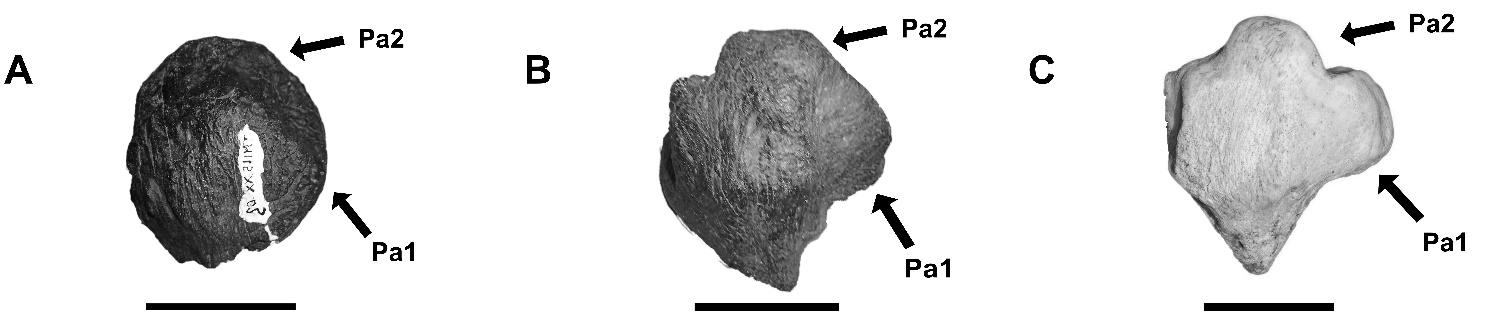


Figure S5. Calcanei of Hippopotamoids from TM and extant common hippopotamus.

A, *Libycosaurus bahri* (TM9-00-14, right) in distal (A) view. B*, Hexaprotodon garyam* (TM335-05-04, left, mirrored) in distal (B) view. C*, Hippopotamus amphibius* (OST-361, left, mirrored) in distal (C) view. Scale bars = 5 cm.


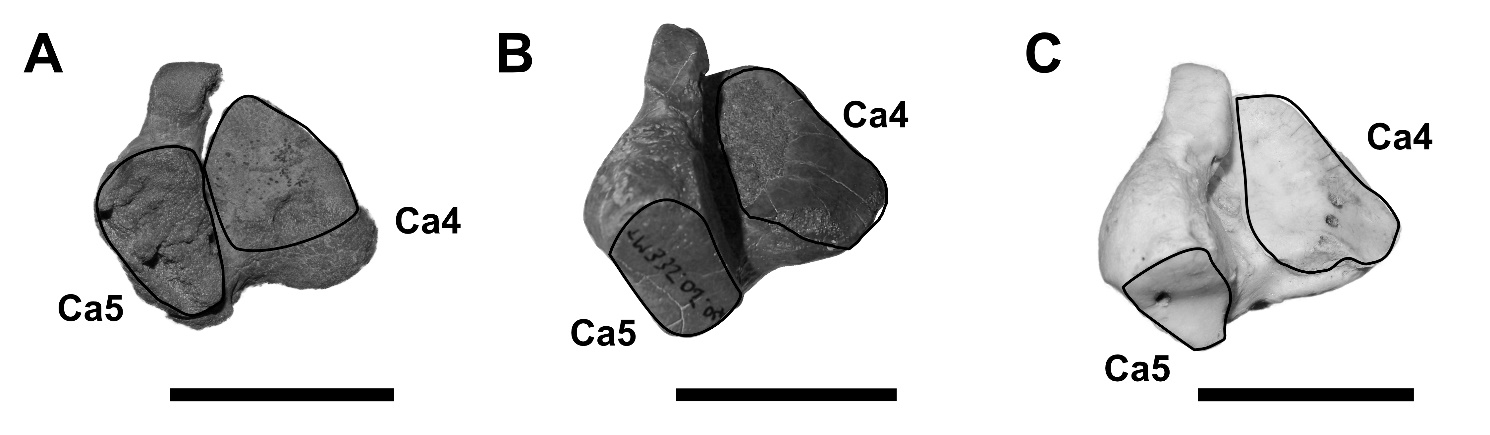


Figure S6. Distal row of tarsal bones of Hippopotamoids from TM and extant common hippopotamus.

A-B, Cuboid; *Libycosaurus bahri* (TM259-08-07, left, mirrored) in lateral (A) and cranial (B) views. C-D*,* Cuboid; *Hexaprotodon garyam* (TM361-02-04, , left, mirrored) in lateral (C) and cranial (D) views. E-F*,* Cuboid; *Hippopotamus amphibius* (HIPPO-ACTU, right) in lateral (E) and cranial (F) views. Scale bars = 5 cm. G-H, Navicular; *Libycosaurus bahri* (TM254-02-08, left) in lateral (G), distal (H) views. I-J*,* Navicular; *Hexaprotodon garyam* (TM171-01-15, right, mirrored) in lateral (I), distal (DJ views. K-L*,* Navicular; *Hippopotamus amphibius* (MNHN-1897-33, left) in lateral (K), distal (L) views. Scale bars = 5 cm.


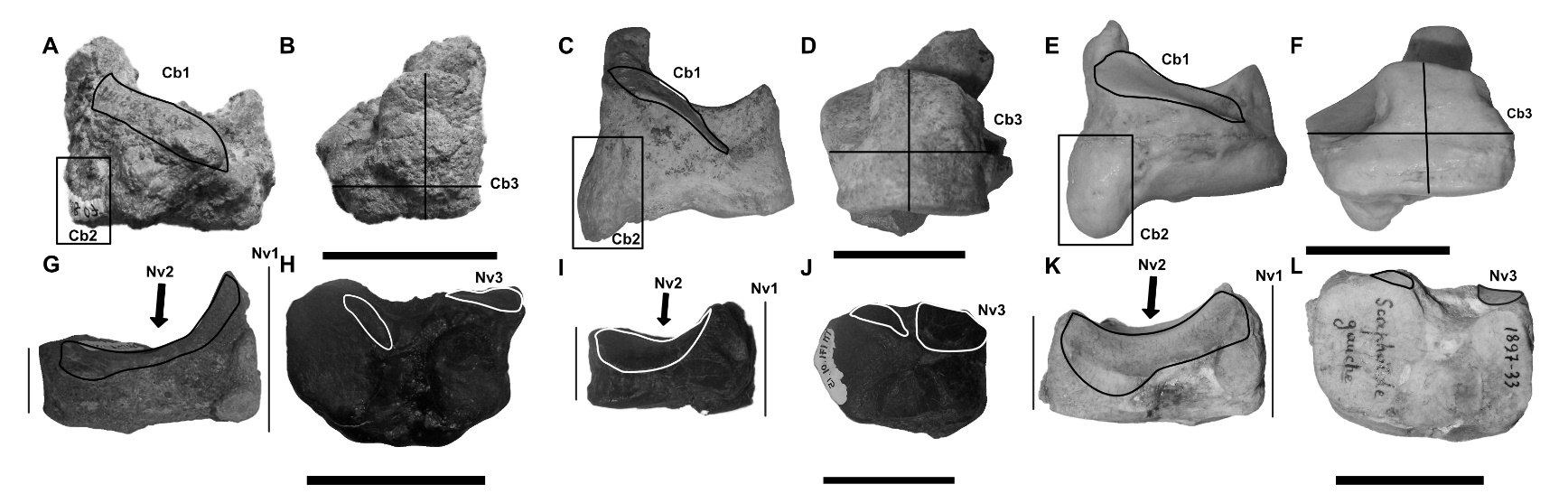


Figure 16. External metacarpals of Hippopotamoids from TM and extant common hippopotamus.

A-D, MC II; *Libycosaurus bahri* (TM115-00-041, mirrored) in cranial (A), palmar (B), proximal (C) and distal (D) views*.* E-H*,* MC II; *Hexaprotodon garyam* (TM112-00-86) in cranial (E), palmar (F), proximal (G) and distal (H) views*.* I-L*,* MC II; *Hippopotamus amphibius* (MNHN-1897-33) in cranial (I), palmar (J), proximal (K) and distal (L) views*.* Scale bars = 5 cm*.* M-N, MC V; *Libycosaurus bahri* (TM259-08-06, mirrored) in palmar (M), medial (N) and distal (O) views*.* O-P*,* MC V; *Hexaprotodon garyam* (TM09-01-328, mirrored) in palmar (P), medial (Q) and distal (R) views*.* Q-R*,* MC V; *Hippopotamus amphibius* (MNHN-1897-33; MNHN-1917-249, mirrored for T) in palmar (S), medial (T) and distal (U) views*.* Scale bars = 5 cm.

Figure S7. External metacarpals of Hippopotamoids from TM and extant common hippopotamus.

A-D, MC II; *Libycosaurus bahri* (TM115-00-041, right) in cranial (A, mirrored), palmar (B, mirrored), proximal (C) and distal (D) views*.* E-H*,* MC II; *Hexaprotodon garyam* (TM112-00-86; left) in cranial (E, mirrored), palmar (F), proximal (G) and distal (H, mirrored) views*.* I-L*,* MC II; *Hippopotamus amphibius* (MNHN-1897-33, left) in cranial (I), palmar (J), proximal (K) and distal (L) views*.* Scale bars = 5 cm*.* M-N, MC V; *Libycosaurus bahri* (TM259-08-06, left) in palmar (M, mirrored), medial (N) and distal (O) views*.* O-P*,* MC V; *Hexaprotodon garyam* (TM09-01-328, left) in cranial (P, mirrored), medial (Q) and distal (R) views*.* Q-R*,* MC V; *Hippopotamus amphibius* (MNHN-1897-33, left; MNHN-1917-249, right for T) in cranial (S), medial (T, mirrored) and distal (U) views*.* Scale bars = 5 cm.
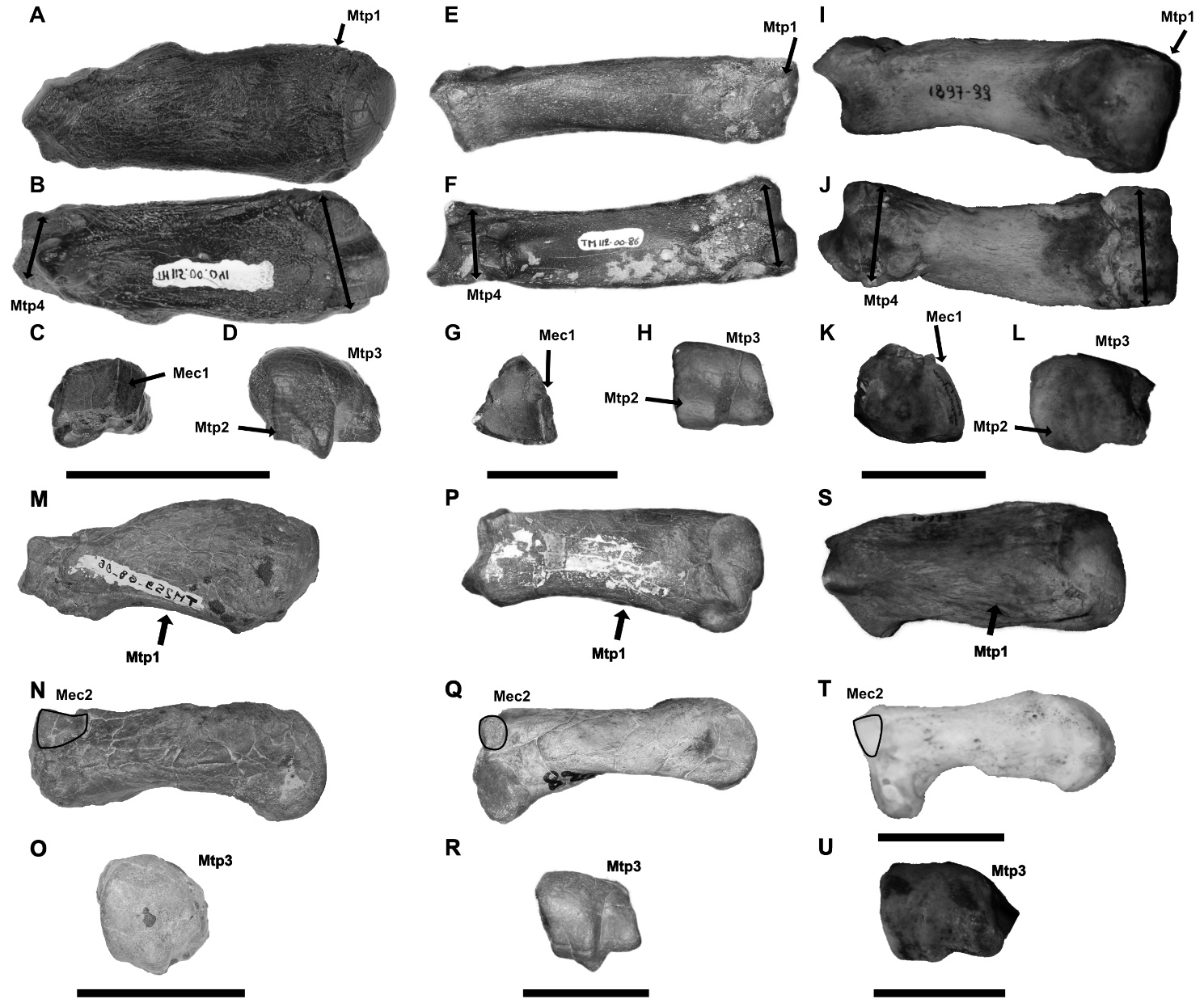


Figure S8. External metatarsals of Hippopotamoids from TM and extant common hippopotamus.

A-C, MT II; *Libycosaurus bahri* (TM133-15-14, left) in cranial (A, mirrored), palmar (B, mirrored) and distal (C) views*.* D-F*,* MT II; *Hexaprotodon garyam* (TM09-01-207) in cranial (D), palmar (E) and distal (F) views*.* G-I*,* MT II; *Hippopotamus amphibius* (MNHN-1924-134, mirrored) in cranial (G), palmar (H) and distal (I) views*.* Scale bars = 5 cm*.* J-L, MT V; *Libycosaurus bahri* (TM299-08-018, right) in medial (J, mirrored), proximal (K) and distal (L) views*.* M-O*,* MT V; *Hexaprotodon garyam* (TM115-00-45) in medial (M), proximal (N) and distal (O) views*.* P-R, MT V; *Hippopotamus amphibius* (MNHN-1924-134) in medial (P), proximal (Q) and distal (R) views*.* Scale bars = 5 cm.

*
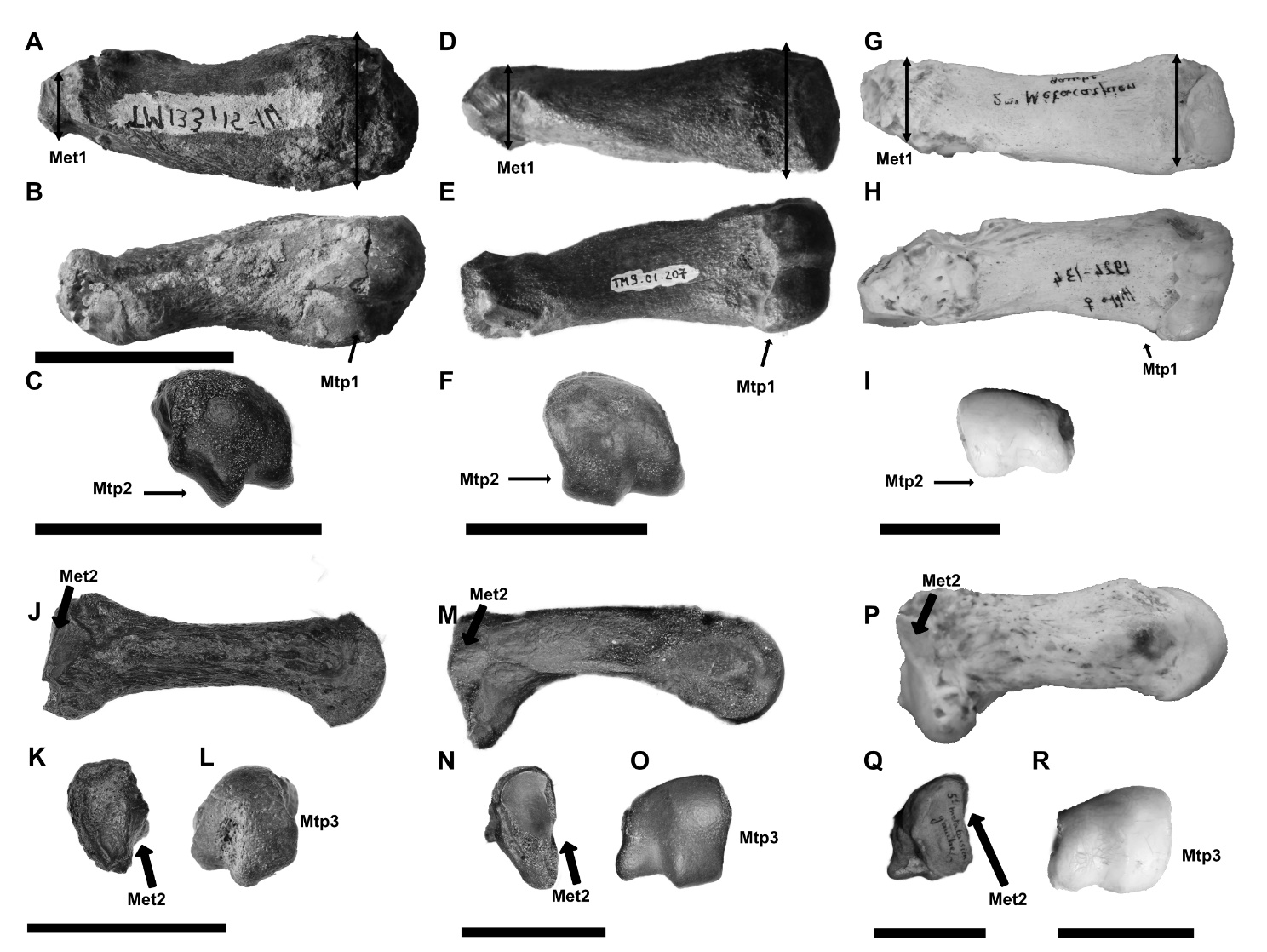
*

Figure S9. Central metatarsals of Hippopotamoids from TM and extant common hippopotamus.

A-E, MT III; *Libycosaurus bahri* (TM379-04-012c, right) in cranial (A), palmar (B), medial (C), lateral (D) and distal (E) views. F-J*,* MT III; *Hexaprotodon garyam* (TM123-01-015, right, mirrored) in cranial (F), palmar (G), medial (H), lateral (I) and distal (J) views. K-O*,* MT III; *Hippopotamus amphibius* (MNHN-1897-33, left) in cranial (K), palmar (L), medial (M), lateral (N) and distal (O) views. Scale bars = 5 cm. P-T, MT IV; *Libycosaurus bahri* (TM123-01-02, right, mirrored) in cranial (P), palmar (Q), medial (R), lateral (S) and distal (T) views. U-Y*,* MT IV; *Hexaprotodon garyam* (TM55-98-07, left) in cranial (U), palmar (V), medial (W), lateral (X, mirrored) and distal (Y) views. Z-D’*,* MT IV; *Hippopotamus amphibius* (MNHN-1897-33, left) in cranial (Z), palmar (A’), medial (B’, mirrored), lateral (C’) and distal (D’) views. Scale bars = 5 cm.


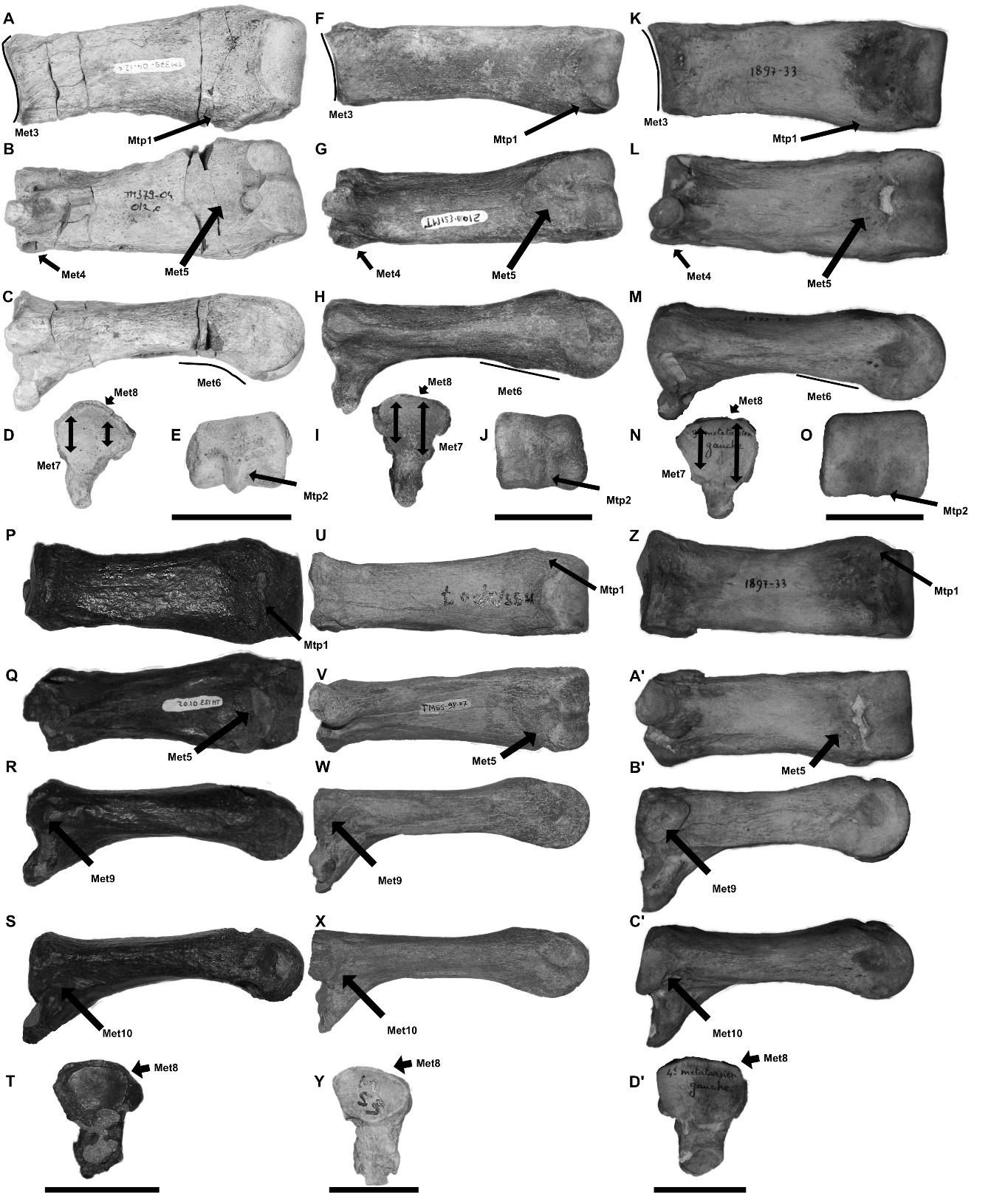

Supplement: Supplementary file 1 — Data S1: Supplementary Figures. [file JOA-9999-0-s003.docx]
