# Supplementary material for: Postcranial anatomy of the Miocene hippopotamoids of Toros‐Menalla, Chad
Source: J Anat. 2026 Mar 19:10.1111/joa.70135. Online ahead of print. doi: 10.1111/joa.70135 (PMC13399167; doi:10.1111/joa.70135)
Supplement: Supplementary file 2 — Data S2: Supplementary Tables. [file JOA-9999-0-s004.docx]

The weight of both species has been estimated from their astragali (following (Martinez & Sudre, 1995). The range from 1,000 kg to 2,200 kg for *Hex. garyam*, averaging 1,500 kg, is very close to the usual weight range for the extant *Hip. amphibius* which is between 1,000 kg and 2,000 kg (Kingdon, 2014). The range shifts downwards by about 500 kg for *L. bahri*, between 500 kg and 1,500 kg with an average of 920 kg. The estimations on the long bones provided different results depending on the measurement used for heavy artiodactyls (Supplementary Material II). Indeed, the estimations based on the stylopod bones and the tibia yielded results consistent with the literature, with *L. bahri* specimens weighing around 500 kg, weight range characteristic of the merycopotamin that is absent in both *Hip. amphibius* and *Hex. garyam.* When using the transverse width of the tibial plateau, the results were also consistent with the literature and previous estimates. However, the results of the estimations based on ulnar, radial, and tibial length measurements (U1, R1 and R4) were abnormal. These ranged from 100 kg to 600 kg for the extant common hippopotamus, showing that the use of the radioulnar and tibial lengths for body mass estimations in hippopotamoids is not relevant. The shortening of the forelimb zeugopodium and the strong curvature of the olecranon in hippopotamoids might explain the abnormal results yielded by the equations based on the radioulna.

Table S1 : Recapitulative tables of body mass estimations from Scott (1983) with respective means, number of specimens and standard deviation for each species. We used seven different measurements from Scott (1983) : H1, U1, R1, F1, and T1, all being the maximum lengths of respectively the humerus, ulna, radius, femur and tibia and R4 and T2, being the transverse widths of the proximal radius and of the tibial plateau, respectively.

| Forelimb | Humerus | | | Radioulna | | | | | | | | |
| --- | --- | --- | --- | --- | --- | --- | --- | --- | --- | --- | --- | --- |
|  | H1 | | | U1 | | | R1 | | | R4 | | |
|  | BM (kg) | n | SD | BM (kg) | n | SD | BM (kg) | n | SD | BM (kg) | n | SD |
| *Hippopotamus amphibius* | 1111.79 | 13 | 267.06 | 294.62 | 11 | 85.78 | 642.10 | 11 | 187.91 | 575.47 | 14 | 99.66 |
| *Hexaprotodon garyam* | 1111.45 | 4 | 171.58 | 339.66 | 9 | 45.43 | 740.70 | 9 | 99.62 | 376.82 | 16 | 149.24 |
| *Libycosaurus bahri* | 939.19 | 4 | 599.76 | 156.37 | 10 | 63.08 | 339.74 | 10 | 137.66 | 266.58 | 18 | 53.43 |

| Hindlimb | Femur | | | Tibia | | | | | |
| --- | --- | --- | --- | --- | --- | --- | --- | --- | --- |
|  | F1 | | | T1 | | | T2 | | |
|  | BM (kg) | n | SD | BM (kg) | n | SD | BM (kg) | n | SD |
| *Hippopotamus amphibius* | 1131.19 | 12 | 280.96 | 269.99 | 11 | 97.92 | 1375.30 | 11 | 345.75 |
| *Hexaprotodon garyam* | 1201.64 | 3 | 56.44 | 261.79 | 21 | 85.20 | 1178.99 | 17 | 240.82 |
| *Libycosaurus bahri* | 673.10 | 4 | 354.94 | 164.35 | 3 | 32.81 | 459.20 | 1 | / |

Scott, K.M. (1983) Prediction of body weight of fossil Artiodactyla. *Zoological Journal of the Linnean Society*, 77, 199–215. Available from: <https://doi.org/10.1111/j.1096-3642.1983.tb00098.x>

Table S2 : Recapitulative tables of body mass estimations from astragalus following Martinez and Sudre, 1995.

|  | Astragalus | | |
| --- | --- | --- | --- |
|  | BM (kg) | n | SD |
| *Hippopotamus amphibius* | 1640,27 | 4 | 662,10 |
| *Hexaprotodon garyam* | 1504,73 | 89 | 240,04 |
| *Libycosaurus bahri* | 1243,48 | 37 | 312,62 |

Martinez, J. & Sudre, J. (1995) The astragalus of Paleogene artiodactyls: comparative morphology, variability and prediction of body mass. *Lethaia*, 28, 197–209. Available from: https://doi.org/10.1111/j.1502-3931.1995.tb01423.x
