# Supplementary material for: Postcranial anatomy of the Miocene hippopotamoids of Toros‐Menalla, Chad
Source: J Anat. 2026 Mar 19:10.1111/joa.70135. Online ahead of print. doi: 10.1111/joa.70135 (PMC13399167; doi:10.1111/joa.70135)
Supplement: Supplementary file 6 — Data S6: SuppMatV_Suid_Comparative_Description. [file JOA-9999-0-s006.docx]

**IV. Bone by bone comparison of the suid *Sus scrofa* and the three Hippopotamoidea**

1. Axial skeleton

1.1. Atlas

*Sus scrofa* differs quite largely from the hippopotamoids studied here by a deep and wide cranial notch (At1) and a medio-laterally elongated and cranio-ventrally narrow caudal articular facets with the axis (At5).

1.2. Axis

The prezygapophyses (Ax4) of *S. scrofa* are elongated medio-laterally and flat cranio-caudally which is never the case in the studied hippopotamoid. The neural arches are also very thin in *Sus* and the axis is more cranio-caudally compressed.

*S. scrofa* differs from *Hippopotamus* and *Libycosaurus* by a lateral development of the processes and apophyses that is less marked, as is the development of the neural spines.

2.1. Scapula

The common boar scapula differs from those of hippopotamoids as it display a strong acromion process (Sp2) and a poorly developed supraglenoid tubercle, forming a small tuberosity close to the cranial edge of the glenoid cavity (Sp3).

3.1. Humerus

In proximal view, the non-articular area between the caudal-most point of the humeral head and the cranial-most point of the great trochanter of *S. scrofa* is larger than in the three hippopotamoids. On the diaphysis, no deltoid tuberosity is easily observable in the boar.

3.2. Radioulna

Unlike in the three hippopotamoids, the radius and ulna of *S. scrofa* are unfused. It also differs on the olecranon, by the lack of visible medial extension and on the radius, by the distal articular surface where the contact facet with the scaphoid is relatively larger and rounder and the pyramidal facet is proportionally smaller.

4.1. Scaphoid

A small articular facet with the semilunar is clearly visible laterally in the boar where it is absent or incipient in the hippopotamoids.

4.2. Semilunar

In *S. scrofa*, the distal articular surface displays two contact facets with the magnum, while hippopotamoids only display one.

4.3. Pyramidal

In the wild boar, a strongly developed medial articular facet contacts the semilunar. This facet is less prominent in hippopotamoids. Also, the contact facet with the pisiform is relatively much larger in hippopotamoids than in *S. scrofa*.

4.4. Magnum

In *S. scrofa*, the magnum is much less projected palmarly. Moreover, at least two lateral articular facets articulating with the unciform are visible whereas only one, more expanded, is present in hippopotamoids.

4.5. Unciform

As for the magnum, the unciform of *S. scrofa* displays at least two medial articular facets but only one in the three hippopotamoids.

5.1. Femur

Femurs of wild boars do not display the deep supracondylar fossa that is present in the three studied hippopotamoids. Moreover, their femurs are much more gracile. The femoral neck and head are also relatively larger and thicker in *S. scrofa*, with the additional presence of the *fovea capitis*, absent in the hippopotamoids.

5.3. Tibia

The tibial crest is much stronger relatively to the overall robustness of the bone in the hippopotamoids than in *S. scrofa.* On the distal articular surface with the astragalus, the surface is clearly divided by a strong crest in the wild boar, crest that is flattened and less marked in the hippopotamoids.

6.1. Astragalus

The astragalus of *S. scrofa* is much more compressed medio-laterally than those of the three hippopotamoids. Distally, the articular surface with the distal row of the tarsals is more asymmetrical (with a smaller facet for the cuboid) than in the astragali of hippopotamoids. It also displays more marked keels and trochlea and the synovial fossa is absent. Moreover, the sustentacular facet is concave in hippopotamoids and convex in the suid.

6.2. Calcaneus

The sustentacular process of the calcaneus of *S. scrofa* is straight and does not exhibit a particular division of the contact facets with the plantar articular facet of the astragalus like in the hippopotamoids. The sustentacular facet is concave and oval-shaped in opposition to the convex and triangular morphology it displays in hippopotamoids. Additionally, the plantar tubercle is relatively thinner in suids, and the cuboid facet is concave, whereas it is convex in hippopotamoids.

6.3. Cuboid

The cuboid of the wild boar is more compressed medio-laterally than in the hippopotamoids. There is only one large plantar facet in suids in contrary to the two plantar facets present in the studied hippopotamoids. Additionally, the wild boar posterior-most plantar facet is on a marked plantar process. This process is non-articular in hippopotamoids.

6.4. Navicular

The navicular displays a disto-plantar process in *S. scrofa*, which is absent in the studied hippopotamoids. This process is articular and thus presents a facet that allows a close articulation with the cuboid, as stated previously.

7.1. Second and fifth metacarpals

In *S. scrofa*, the second metacarpal is much thinner relatively than in the hippopotamoids. Its distal articular surface is strongly asymmetrical, and its distal cross-section is triangular as opposed to the squared or rounded cross section of hippopotamoids.

For this metapodial (V), the same characters are visible than what was previously cited for the second metacarpal.

7.2. Third and fourth metacarpals

The wild boar displays a strongly projected lateral process contacting the fourth metacarpal and the unciform, with a marked concavity between the lateral and palmar process in proximal view. This process is less projected in hippopotamoids, and the concavity is reduced. More importantly, the boar displays a medial articular facet contacting the trapezoid bone. This contact is completely absent in the three hippopotamoids studied. Additionally, the distal articular surface is strongly asymmetrical in *S. scrofa*, with a marked distal keel that extends on both anterior and palmar sides, which is not the case in the hippopotamoids (the moderate to weak keel is restricted to the palmar side).

The fourth metacarpal displays the same distal articular surface as the third one in *S. scrofa*. Additionally, the proximal articular surface exhibits a marked notch on its medial side, corresponding to the lateral process of the MC III. This notch is narrower in the hippopotamoids.

7.3. Second and fifth metatarsals

The second metatarsal of the wild boar displays the same general characters as the external metacarpals, as in displaying a triangular cross-section and a very reduced proximal articular surface.

The fifth metatarsal of the wild boar displays the same general characters as the external metacarpals, as in displaying a triangular cross-section and a very reduced proximal articular surface.

7.4. Third and fourth metatarsals

The third metatarsal follows the trend of the third metacarpals, especially in terms of asymmetry of the distal articular surface and presence of a strong continuous keel along this same surface.

The fourth metatarsal follows the trend of the metacarpals, especially in terms of asymmetry of the distal articular surface and presence of a strong continuous keel across all this same surface.
